# mTOR signaling contributes to system-driven rhythmic gene expression in mouse liver

**DOI:** 10.1126/sciadv.aec0131

**Published:** 2026-07-23

**Authors:** Aishwarya Sahasrabudhe, Chanté R. Guy, Audrey Jacq, Chieh-Wen Ho, Ben J. Greenwell, Jerome S. Menet

**Affiliations:** ^1^ Department of Biology, Center for Biological Clocks Research, Texas A&M University, College Station, TX 77843, USA.; ^2^ Program of Genetics, Texas A&M University, College Station, TX 77843, USA.

## Abstract

Rhythmic gene expression is essential to the daily organization of biological processes. While cycling transcriptomes are regulated by circadian clocks present in nearly every cell, accumulating evidence indicates that they can also be initiated by rhythmic food-driven systemic signals independently of circadian clocks. The underlying mechanisms remain however largely unknown. Here, we show that signaling through the nutrient-sensing kinase mechanistic target of rapamycin (mTOR) is both necessary and sufficient to mediate food-driven hepatic rhythmic gene expression, rhythmic regulation of the liver metabolome, and endoplasmic reticulum stress response. Acute inhibition of mTOR before the active phase desynchronizes the phase of mTOR-driven rhythmic genes without affecting clock-controlled rhythmic genes, indicating that alignment of rhythmic mTOR activity to the circadian cycle is critical for overt cycling transcriptomes. These findings may explain how misalignment between clock and systemic signals contributes to disease and underscore the use of mTOR inhibitors for resynchronizing system-driven rhythms and alleviating circadian rhythm disorders.

## Teaser

Nutrient signals regulate the cycling liver transcriptome through oscillating mTOR activity.

## INTRODUCTION

The activity of most biological processes oscillates across the 24-hour day, enabling organisms to anticipate and adapt to daily changes in the environment. Much of this temporal regulation occurs at the transcriptional level, with thousands of genes encoding for enzymes, receptors, transporters, transcription factors (TFs), and other cellular effectors exhibiting daily rhythms in expression in nearly every tissue (*1*–*4*). At the core of this regulation lies a timekeeping mechanism, the circadian clock, that consists of interlocked transcriptional/translational feedback loops (*5*). In mammals, the circadian clock is initiated by the heterodimeric TFs CLOCK:BMAL1 and NPAS2:BMAL1, which bind DNA during the day and drive the rhythmic expression of the transcriptional repressors *Period* (*Per1*, *Per2*, and *Per3*) and *Cryptochrome* (*Cry1* and *Cry2*) genes. PERs and CRYs inhibit CLOCK:BMAL1- and NPAS2:BMAL1-mediated transcription at night before being degraded to reinitiate a new cycle of transcription. An auxiliary feedback loop involving the TFs *Ror* (*Ror*α, *Ror*β, and *Ror*γ) and *Rev-erb* (*Rev-erb*α and *Rev-erb*β, also known as *Nr1d1* and *Nr1d2*, respectively) reinforces clock oscillation by activating or repressing *Bmal1* and *Clock* transcription, respectively. In addition to regulating clock gene expression, the circadian clock drives rhythmic transcription of thousands of genes that are commonly referred to as clock-controlled genes (*6*). Mammalian circadian clocks are present in nearly every cell across the body where they regulate tissue-specific biochemical, physiological, and metabolic rhythms (*7*–*9*). These so-called peripheral clocks are coordinated by the master circadian pacemaker located in the suprachiasmatic nucleus (SCN) of the hypothalamus, which is entrained to the environmental light-dark cycle via retinal innervation through the retinohypothalamic tract (*10*, *11*). SCN-driven rhythms in neuronal and hormonal signals, feeding, temperature, and other physiological cues coordinate peripheral clocks across the body (*12*–*17*).

While cycling transcriptomes are regulated locally by the circadian clock that is present within each cell, increasing evidence indicates that rhythmic gene expression can also emerge as a response to rhythmic extracellular signals, independently of the molecular clock oscillations (*16*, *18*–*21*). Among these, the daily rhythm of food intake appears particularly relevant. For example, clock-deficient knockout mice fed rhythmically exhibit rhythmic expression of hundreds of hepatic genes despite the absence of a functioning circadian clock (*18*–*21*). In addition, wild-type (WT) mice that consume their daily calorie intake equally across the 24-hour day exhibit a substantial loss of rhythmic gene expression without any effect on the hepatic molecular clock rhythms (*19*). However, the mechanisms through which rhythmic food intake (RFI) drives rhythmic gene expression remain poorly defined.

The protein mechanistic target of rapamycin (mTOR) is an evolutionary conserved serine/threonine kinase that is responsive to various intra- and extracellular signals including nutrients, energy status, insulin, and growth factors. mTOR forms multiprotein complexes that regulate key biological processes such as translation, cell growth, autophagy, glucose and lipid metabolism, and cytoskeletal organization (*22*, *23*). In the mouse liver, mTOR activity is rhythmic and tightly coupled to feeding behavior: It peaks at night in mice fed ad libitum or only during the night but becomes arrhythmic in arrhythmically fed mice (*19*, *24*–*28*).

Given the central role of mTOR in coordinating cellular processes in response to nutrient availability, we hypothesized that mTOR functions as a hub integrating food intake–driven systemic signals to mediate the rhythmic expression of genes that are regulated by the daily rhythm of food intake. Using pharmacological inhibition of mTOR in mice, we found that mTOR is not only necessary but also sufficient to restore most of the rhythms lost upon arrhythmic feeding (AF) in the mouse liver, independent of the hepatic molecular clock. Our results further show that pharmacological inhibition of mTOR activity before the active phase desynchronizes clock- and mTOR-driven rhythmic gene expression, offering insights into mechanisms underlying circadian rhythm desynchronization (CRD). These findings also highlight the importance of aligning rhythmic mTOR activity with the circadian cycle for the maintenance of the rhythmic metabolome and of rhythmic biological functions such as protein processing in the endoplasmic reticulum (ER).

## RESULTS

### Pharmacological inhibition of mTOR decreases rhythmic gene expression in mouse liver independently of the hepatic circadian clock

**Fig. 1.**
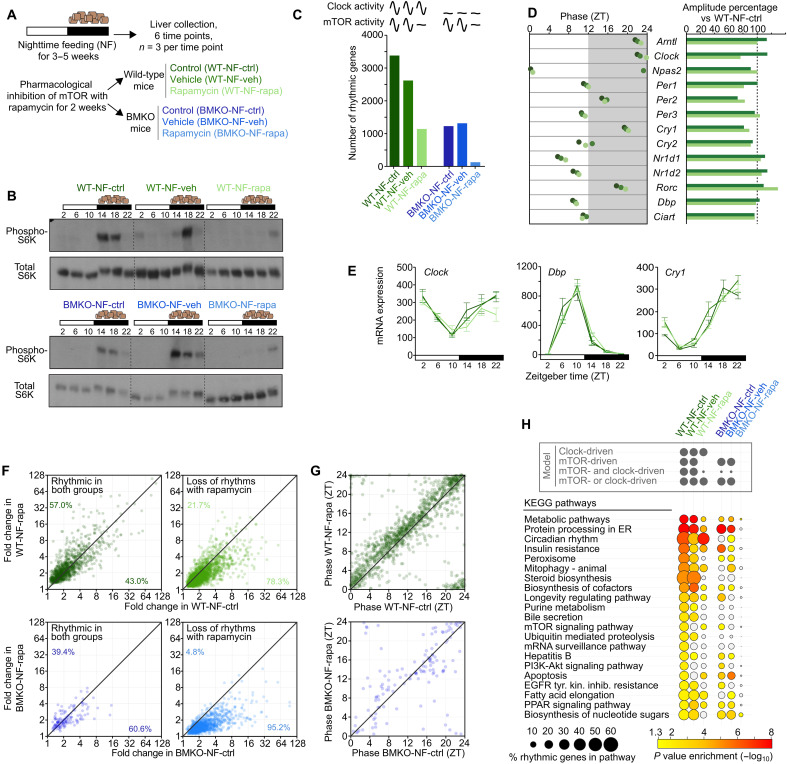
Pharmacological inhibition of mTOR decreases rhythmic gene expression in the mouse liver. (**A**) Experimental design illustrating the different groups of mice and feeding regimens. (**B**) Western blot analysis of phospho-S6K and total S6K levels in the mouse liver. (**C**) Number of rhythmic genes in the mouse liver for each of the six groups of mice (*P*_adj_ ≤ 0.05), calculated using BH-corrected harmonic mean *P* values from four rhythmicity tests: F24, JTK_CYCLE, RAIN, and HarmonicRegression. (**D**) Phase and amplitude (plotted as % of amplitude versus WT-NF-ctrl mice) of clock genes across different mouse groups. (**E**) Gene expression profiles of three clock genes (*Clock*, *Dbp*, and *Cry1*) in the liver of WT-NF-ctrl, WT-NF-veh, and WT-NF-rapa mice. Values represent the means ± SEM; *n* = 3 per time point. (**F**) Analysis of fold change in expression for genes that remained rhythmic (*P*_adj_ ≤ 0.05, left) and those that became nonrhythmic after mTOR inhibition (*P*_adj_ > 0.05, right) in WT (green) and BMKO (blue) mice. Fold change was calculated as the ratio between the mean maximal expression to the mean minimal expression. (**G**) Phase analysis between control and mTOR-inhibited mice for genes that remained rhythmic in WT (green) and BMKO (blue) mice. (**H**) KEGG pathway enrichment analysis of genes that lost rhythmicity upon mTOR inhibition across the different groups. A predictive model for genes coined clock-driven, mTOR-driven, mTOR- and clock-driven, and mTOR- or clock-driven is provided on the top. Selected enriched KEGG pathways are represented with the size of the circle representing the percentage of rhythmic genes in the pathway and the color scale indicating *P* value enrichment (−log_10_).

To determine whether mTOR contributes to 24-hour rhythms in gene expression, we first used a pharmacological approach to inhibit mTOR in vivo with rapamycin and assess its impact on the hepatic cycling transcriptome. We subjected WT mice to a night-restricted feeding (NF) schedule (*19*, *29*) to promote overt food-driven rhythms in gene expression. All mice used in this study had their food intake monitored (fig. S1). Mice were then assigned to one of the following three groups: NF control (WT-NF-ctrl), NF intraperitoneally injected with vehicle (WT-NF-veh), and NF intraperitoneally injected with rapamycin (WT-NF-rapa). Rapamycin did not affect nighttime food intake levels (fig. S1). After 2 weeks, livers were collected every 4 hours over 24 hours (Fig. 1A). To confirm effective mTOR inhibition by rapamycin, we assayed the phosphorylation status of a direct mTOR target, protein ribosomal S6 kinase (S6K), in the mouse liver. As previously reported (*19*, *24*–*28*), mTOR activity exhibited robust rhythmicity, peaking at night coincident with food intake. Rapamycin treatment strongly decreased S6K phosphorylation, indicating effective mTOR inhibition (Fig. 1B and fig. S2, A and B). Transcriptomic analysis revealed that rapamycin reduced the number of rhythmic genes by more than 50% compared to control and vehicle-treated mice (66 and 55%, respectively), suggesting that rhythmic mTOR activity plays a crucial role in driving rhythmic gene expression in the mouse liver (Fig. 1C, fig. S2C, and tables S1 to S3). Rapamycin treatment did not alter the phase or amplitude of core clock gene expression (Fig. 1, D and E), indicating that the effect of rapamycin on the cycling transcriptome is not mediated by the hepatic circadian clock.

To confirm that the circadian clock had little involvement in the rapamycin-mediated reduction in rhythmic gene expression, we replicated the experiment in whole-body *Bmal1^−/−^* (BMKO) mice fed only at night (Fig. 1, A and B, and fig. S2, A and B). Similar to WT mice, rapamycin did not affect nighttime calorie intake in BMKO mice (fig. S1). As shown previously, restricting BMKO mice to nighttime feeding restored rhythmic gene expression, albeit at lower levels than in WT control mice (*18*, *19*, *21*). However, rapamycin treatment markedly reduced this rescue (Fig. 1C and fig. S2, A and B), confirming that RFI-mediated rhythmic gene expression relies on rhythmic mTOR activity and does not require a functional circadian clock.

To evaluate the effect of rapamycin on rhythmic gene expression, we analyzed fold-change expression (amplitude cannot be reliably used for nonrhythmically expressed genes) of genes that remained rhythmic or lost rhythmicity after mTOR inhibition in both WT and BMKO livers (Fig. 1F). Genes that remained rhythmic showed no substantial changes between rapamycin-treated and control groups. In contrast, genes that lost rhythmicity after mTOR inhibition showed a strong decrease in fold-change expression, with more than 75% of these genes showing decreased fold changes relative to control groups (78.3% in WT mice and 95.2% in BMKO mice) (Fig. 1F). Phase analysis of genes that remained rhythmic after mTOR inhibition revealed no phase shift compared to control mice after mTOR inhibition (Fig. 1G).

To explore the functional relevance of these rhythms, we performed KEGG (Kyoto Encyclopedia of Genes and Genomes) pathway enrichment analysis of rhythmic genes across all groups. The pathway “circadian rhythm” remained enriched in all WT groups regardless of mTOR activity status but showed reduced enrichment in BMKO mice, confirming that this pathway is composed of genes whose rhythmicity is primarily clock-driven (Fig. 1H and table S4). By contrast, pathways such as protein processing in the ER and peroxisome were enriched in all mice with rhythmic mTOR activity regardless of clock status, suggesting that these are predominantly mTOR-driven pathways. However, most pathways showed shared dependence on both mTOR and the circadian clock rather than being solely driven by either one alone. Representative genes from some enriched pathways are shown in figs. S3 and S4. This suggests that biological pathways comprise genes whose rhythmicity is not only driven by the molecular clock but also by mTOR rhythmic activity (Fig. 1H).

Together, these results demonstrate that rhythmic mTOR activity is required for driving a large proportion of rhythmic gene expression in the mouse liver in response to rhythmic food intake. This regulation operates independently of the molecular clock in vivo. Moreover, both mTOR- and clock-driven genes converge on similar pathways, underscoring the integrated control of biological rhythms by systemic and molecular timekeeping mechanisms.

### Nutrient inputs to mTOR redundantly regulate rhythmic gene expression

mTOR interacts with several proteins to form two distinct complexes named mTOR complex 1 (mTORC1, scaffolded around RAPTOR) and mTOR complex 2 (mTORC2, scaffolded around RICTOR), which have different upstream inputs and regulate different downstream processes (*22*, *23*, *30*). For example, mTORC1 drives protein synthesis, lipogenesis, and cell growth via phosphorylation of S6K and 4E-BP1 (eukaryotic translation initiation factor 4E binding protein 1), whereas mTORC2 responds to growth factors and stress to regulate cell survival, cytoskeleton, and metabolism via phosphorylation of AKT and SGK1 (serum/glucocorticoid-regulated kinase 1). Rapamycin is a selective inhibitor of mTORC1 (*31*–*33*), yet prolonged rapamycin treatment can also indirectly inhibit mTORC2 assembly and AKT phosphorylation (*34*). To determine whether rapamycin’s impacts on cycling transcripts may involve mTORC2 in addition to mTORC1, we assessed canonical mTORC2 activity by measuring AKT phosphorylation at Ser^473^ (pS473-AKT), a well-established direct target of mTORC2 (*35*, *36*).

Under our experimental setup, AKT phosphorylation at Ser^473^ did not display robust or consistent rhythmicity, particularly when compared to phosphorylation of S6K (fig. S5, A to C). In WT mice, we observed a modest trend toward higher pS473-AKT levels at dusk/night, in line with previous reports (*20*, *25*, *37*), and statistically significant rhythmicity was only detected in rapamycin-treated WT mice. In BMKO mice, pS473-AKT was rhythmic in control and vehicle-treated animals but not in rapamycin-treated mice. The absence of robust rhythmic pS473-AKT in WT mice under our experimental conditions appears largely attributable to substantial interindividual variability, which limits our ability to draw definitive conclusions regarding the impact of pharmacological mTORC1 inhibition on mTORC2 activity (fig. S5, A to C).

**Fig. 2.**
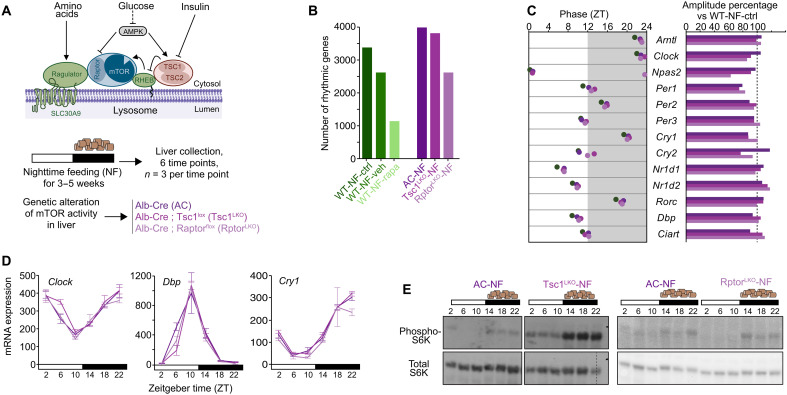
Inputs to mTOR redundantly regulate rhythmic gene expression. (**A**) Nutrient input pathways of mTORC1 (top) and schematic of the experimental design aiming at genetically inhibiting mTORC1 activity (bottom). (**B**) Number of rhythmic genes in the mouse liver (*P*_adj_ ≤0.05). (**C**) Phase and amplitude (plotted as % of amplitude versus WT-NF-ctrl mice) of clock genes across different mouse groups. (**D**) Gene expression profiles of three clock genes (*Clock*, *Dbp*, and *Cry1*) in the liver of AC-NF, *Tsc1^LKO^*-NF, and *Rptor^LKO^*-NF mice. Values represent the means ± SEM; *n* = 3 per time point. (**E**) Western blot analysis of phospho-S6K and total S6K levels in the mouse liver. Western blots for AC-NF and *Tsc1^LKO^*-NF mice have the same exposure time (see fig. S6C).

mTORC1 activation is mediated by RHEB, a lysosomal-anchored small guanosine triphosphatase that binds to mTOR and allosterically activates its kinase activity (Fig. 2A). RHEB is strongly inhibited by the tuberous sclerosis complex (TSC; composed of the proteins TSC1, TSC2, and TBC1D7), particularly when insulin levels are low. By contrast, an increase in amino acid availability activates the Ragulator complex, which recruits mTORC1 to the lysosomal surface through RAPTOR (*38*). In addition, a low glucose level activates AMPK (adenosine 5′-monophosphate–activated protein kinase), which phosphorylates both RAPTOR and TSC2 to ultimately inhibit mTORC1 activity (Fig. 2A). Thus, we next leveraged liver-specific knockouts of *Raptor*, which blocks signaling from amino acid inputs to mTORC1, and *Tsc1*, which uncouples mTORC1 from regulation by insulin signaling, to complement our pharmacological approach and assay the relative contribution of these two inputs to the regulation of rhythmic gene expression by RFI and mTOR.

Liver-specific knockouts of *Tsc1* (*Alb-Cre*; *Tsc1^flox^* mice; abbreviated hereafter *Tsc1^LKO^*; fig. S6A) and *Raptor* (*Alb-Cre*; *Raptor^flox^* mice; abbreviated hereafter *Rptor^LKO^*; fig. S6B) were generated and fed only at night alongside with *Alb-Cre* control mice and analyzed for hepatic rhythmic gene expression (Fig. 2A). Effects of *Tsc1* and *Rptor* knockout on food intake were minimal and restricted to a reduced nighttime intake in *Rptor^LKO^* mice compared to *Alb-Cre* and *Tsc1^LKO^* mice when fed ad libitum (fig. S1). Unexpectedly, neither *Tsc1^LKO^* nor *Rptor^LKO^* had a strong impact on the number of cycling genes (Fig. 2B, fig. S2C, and tables S1 to S3). While *Rptor^LKO^*-NF mice exhibited a small decrease relative to *Tsc1^LKO^*-NF and *Alb-Cre*-NF control mice, this reduction was far less than that observed with rapamycin treatment. These results suggest that amino acid inputs may contribute more potently than insulin signaling to RFI-driven rhythmic gene expression but also highlight the redundancy within the mTOR signaling network, where loss of one input can be compensated by others. Consistent with sustained rhythmic gene expression, core clock oscillations were unaltered in both *Tsc1^LKO^* or *Rptor^LKO^* models (Fig. 2, C and D).

Analysis of phospho-S6K levels supported this notion, as *Tsc1^LKO^* and *Rptor^LKO^* mice exhibited rhythmic S6K phosphorylation with a peak at night similar to AC-NF mice (Fig. 2E and fig. S6, C and D). As expected from the loss of a negative regulator, *Tsc1^LKO^*-NF mice showed elevated levels of phospho-S6K (i.e., higher mTOR activity) across all time points yet maintained the rhythmic pattern, indicating that rhythmic mTOR activity can be maintained through other upstream inputs even under chronically elevated baseline levels (Fig. 2C and fig. S6, C and D). S473 AKT phosphorylation, a marker of mTORC2 activity, was rhythmic with a peak at night in control *Alb-Cre* mice, consistent with the literature (*20*, *25*, *37*), as well as in *Rptor^LKO^* mice. In contrast, pS473 AKT levels were low and arrhythmic in *Tsc1^LKO^* mice, indicating that knocking out *Tsc1* in the liver impairs mTORC2 activity (fig. S5). Given that this blunted mTORC2 activity had limited effects on the cycling hepatic transcriptome (Fig. 2B), this suggests that rhythmic mTOR-dependent outputs seem to be more closely associated with mTORC1 activity than with canonical mTORC2 signaling.

### Resetting mTOR activity by acute inhibition is sufficient to drive rhythmic gene expression in mouse liver

**Fig. 3.**
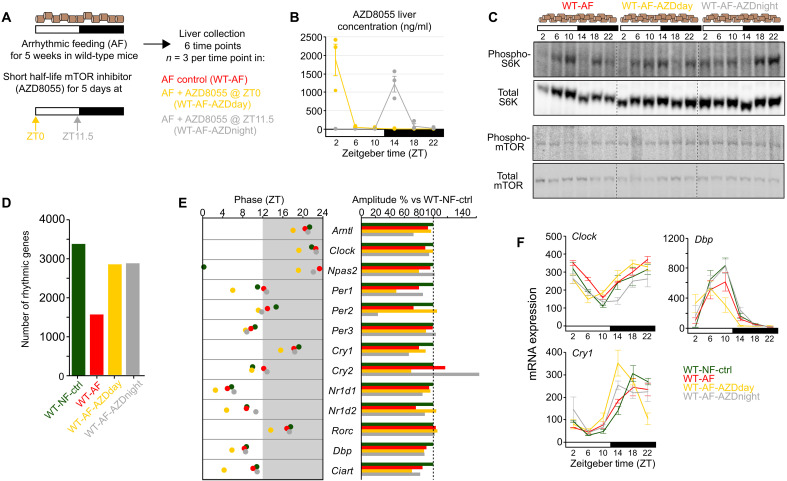
The short half-life mTOR inhibitor AZD8055 rescues rhythmic gene expression in arrhythmically fed WT mice. (**A**) Schematic of the experimental design showing the different groups of mice and feeding regimens. (**B**) AZD8055 concentration in the liver of WT-AZDday and WT-AZDnight mice. (**C**) Western blot analysis of phospho-S6K, total S6K, phospho-mTOR, and total mTOR levels in the mouse liver. (**D**) Number of rhythmic genes in the mouse liver (*P*_adj_ ≤ 0.05). (**E**) Phase and amplitude (plotted as % of amplitude versus WT-NF-ctrl mice) of clock genes across different mouse groups. (**F**) Gene expression profiles of three clock genes (*Clock*, *Dbp*, and *Cry1*) in the liver of WT-NF-ctrl, WT-AF, WT-AF-AZDday, and WT-AF-AZDnight mice. Values represent the means ± SEM; *n* = 3 per time point.

We next asked whether rhythmic mTOR activity is sufficient to initiate rhythmic gene expression in the mouse liver. To this end, we used WT mice fed arrhythmically, a condition previously shown to result in arrhythmic mTOR activity across the day as per the phospho-mTOR signal (*19*). We then aimed to rescue rhythmic mTOR activity pharmacologically by treating mice with a short half-life mTOR inhibitor (Fig. 3A). Because rapamycin has a long half-life (*39*), we used AZD8055, which is a potent and selective adenosine 5′-triphosphate (ATP)–competitive mTOR inhibitor with a short half-life of ∼4 hours (*40*, *41*). We intraperitoneally injected WT mice fed arrhythmically with AZD8055 at either zeitgeber time 0 (ZT0) (WT-AF-AZDday) or ZT11.5 (WT-AF-AZDnight) to establish an antiphasic mTOR inhibition (Fig. 3A). AZD8055 administration transiently reduced food intake for a few hours postinjection (fig. S1). This effect was short-lived and did not change the total daily intake or the overall day/night feeding pattern relative to AF controls.

We confirmed AZD8055 short half-life by high-performance liquid chromatography analysis in the mouse liver, with the drug being detected for ∼2 to 4 hours postinjection under both conditions (Fig. 3B). Western blot analysis further validated mTOR inhibition by AZD8055 in the mouse liver, with reduced phospho-S6K and phospho-mTOR levels, as well as faster-migrating total S6K bands (reflecting increased dephosphorylation of S6K) that were consistently observed at the time point immediately following AZD8055 administration (2 hours postinjection) and returning to baseline levels by 6 hours (Fig. 3C and fig. S7, A to C). This acute mTOR inhibition did not impose sinusoidal rhythms of S6K phosphorylation, likely precluding the detection of statistically significant rhythmicity by standard rhythm-detection algorithms.

Consistent with our previous findings (*19*), mice fed arrhythmically (WT-AF) showed a substantial reduction in the number of rhythmic genes in the mouse liver compared to WT-NF-ctrl mice (Fig. 3D). Notably, both AZD-day and AZD-night injections restored rhythmic gene expression to levels comparable to WT-NF-ctrl mice (Fig. 3D, fig. S2C, and tables S1 to S3), indicating that acute mTOR inhibition is sufficient to restore rhythmic gene expression in AF mice. Although AZD8055 transiently reduced food intake, prior work shows that low-amplitude feeding rhythms resemble AF at the transcriptomic level (*19*). Thus, this transient and low-amplitude reduction in food intake following AZD8055 injection is unlikely to account for the robust restoration of rhythmic gene expression observed here, although a minor contribution cannot be formally excluded. Instead, these findings support a primary role for AZD8055-mediated pharmacological resetting of mTOR activity. This restoration of rhythmic gene expression was largely independent of the circadian clock, as AZD8055 treatment had a minimal impact on the amplitude of core clock gene expression, as exemplified by the amplitude of WT-AF, AZD-day, and AZD-night (Fig. 3E) and *Clock*, *Dbp,* and *Cry1* expression profiles (Fig. 3F), which were largely comparable to WT-NF-ctrl. Moreover, the modest changes in some clock gene mRNA levels in AF mice are insufficient to account for the substantially larger differences detected in rhythmic gene expression across the transcriptome. However, AZD8055 injections at ZT0 advanced the phase of core clock genes by ∼4 hours, whereas injections at ZT11.5 had no effect on the phase (Fig. 3, E and F). Together, these results demonstrate that AZD8055 administration produces a transient and time-locked inhibition of mTOR signaling that is sufficient to initiate rhythmic gene expression in the liver.

### Inhibition of mTOR at its activity peak delays mTOR-driven but not clock-driven rhythmic genes

**Fig. 4.**
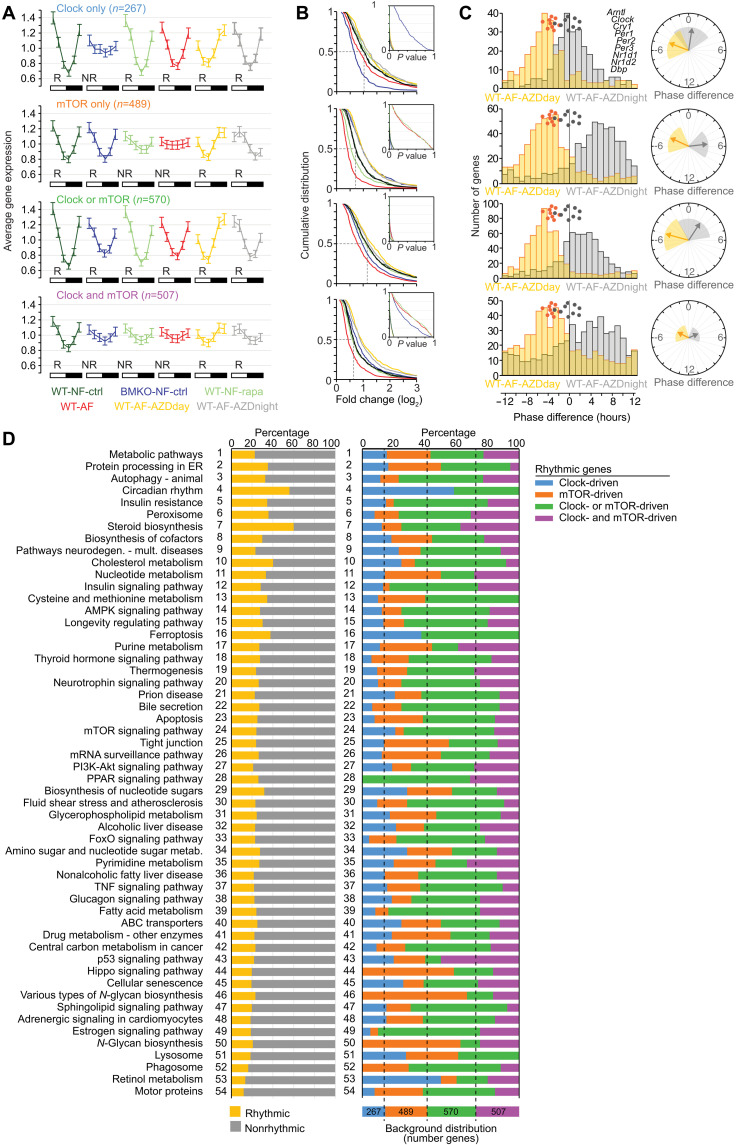
AZD8055 can dissociate the phase of expression of mTOR-driven rhythmic genes versus clock-driven rhythmic genes. (**A**) Average gene expression profile of clock-driven, mTOR-driven, clock- or mTOR-driven, and clock- and mTOR-driven rhythmic genes in different groups of mice (WT-NF-ctrl: dark green; BMKO-NF-ctrl: blue; WT-NF-rapa: light green; WT-AF: red; WT-AF-AZDday: yellow; WT-AF-AZDnight: gray). Values represent the means ± SEM; *n* = 3 per time point. RNA-seq data were grouped, normalized, and aligned as described in Materials and Methods. (**B**) Cumulative distribution of fold change in gene expression (log_2_ scale) for the same six groups of mice. The cumulative distribution of *P* values is shown in the inlet. (**C**) Left: Phase difference between WT-NF-ctrl and WT-AF-AZDday mice (yellow) and between WT-NF-ctrl and WT-AF-AZDnight mice (gray) for clock-driven, mTOR-driven, clock- or mTOR-driven, and clock- and mTOR-driven rhythmic genes. Negative values represent a phase advance in AZD8055-treated mice, while positive values represent a phase delay. The yellow and gray dots represent core clock genes as labeled. Right: Phase difference represented in a circular plot. The length of the arrow represents the concentration of the data point toward the average. The shaded area represents the average +/− 1 standard deviation. (**D**) Left: Percentage of rhythmic genes within different KEGG pathways. Right: Percentage distribution of clock-driven, mTOR-driven, clock- or mTOR-driven, and clock- and mTOR-driven rhythmic genes among the rhythmic genes of different KEGG pathways. The background distribution, which corresponds to the relative number of genes between the four groups, is shown for reference at the bottom of the panel.

To further define how mTOR activity regulates cycling transcriptomes, we classified genes into four distinct categories on the basis of their rhythmicity profiles across experimental groups. Two different thresholding methods were applied, resulting in a stringent gene set and a larger set (see Materials and Methods for details). Unless otherwise noted, results below are presented using the larger gene set as both methods yielded consistent conclusions (fig. S8, A and B). A total of 267 rhythmic genes were classified as solely “clock-driven” for being rhythmic in all experimental groups with an intact circadian clock, irrespective of feeding regime or mTOR activity (i.e., rhythmic in WT-NF-ctrl, WT-NF-rapa, WT-AF, WT-AF-AZDday, and WT-AF-AZDnight but not in BMKO-NF-ctrl mice) (Fig. 4, A and B; fig. S8, A to C; and table S5). Another 489 rhythmic genes were classified as solely “mTOR-driven,” as their rhythmicity was lost specifically in WT-AF and WT-NF-rapa mice, indicating dependence on rhythmic mTOR activity (Fig. 4, A and B, and fig. S8, A to C). In addition, 570 genes were classified as “clock- or mTOR-driven” for being rhythmic in all groups regardless of clock or mTOR status. Last, 507 genes were classified as “clock- and mTOR-driven,” as they lost rhythmicity in all groups where either mTOR or circadian clock oscillations were abolished, i.e., in WT-NF-rapa, WT-AF, and BMKO-NF-ctrl mice (Fig. 4, A and B, and fig. S8, A to C).

To cross-validate our analysis, we leveraged an experimental framework that disentangled the relative contributions of feeding and the circadian clock to rhythmic gene expression by analyzing the liver transcriptome of WT, BMKO, and liver-specific *Bmal1* rescue (liver-RE) mice fed either ad libitum or only at night (*42*, *43*). Notably, the rhythmicity of mTOR-driven genes was markedly enhanced by nighttime feeding, with stronger rhythms observed in time-restricted fed mice compared to ad libitum–fed mice, and was only minimally influenced by the circadian clock, as evidenced by the small differences between WT, BMKO, and *Bmal1*-rescue livers under night-feeding conditions (fig. S9). In contrast, the rhythmicity of clock-driven genes was robust and comparable between WT and liver-RE mice, substantially reduced in BMKO mice, and only weakly affected by the feeding regimen. Together, these results validate our gene categorization and support the conclusion that mTOR-driven rhythmic gene expression is primarily shaped by feeding-related cues rather than the core circadian clock.

Phase analysis revealed that AZD8055 injection at ZT0 advanced the phase of gene expression relative to WT-NF-ctrl mice by ∼4 hours across all four gene categories (Fig. 4C), consistent with the advanced phase observed in core clock genes (Fig. 3E). Conversely, AZD8055 injection at ZT11.5 did not alter the phase of clock-driven genes, aligning with the lack of effects on core clock gene expression (Fig. 3E). However, AZD-night injections delayed the phase of mTOR-driven genes by ∼5 hours (Fig. 4C and fig. S8B), suggesting that mTOR inhibition just before the active phase and its peak of activity can uncouple the phase of mTOR-driven genes from clock-driven genes. Clock- or mTOR-driven genes showed an intermediate phase shift, more closely resembling clock-driven genes, whereas clock- and mTOR-driven genes exhibited a similar ∼4-hour phase delay to mTOR-driven genes, although with greater variability in timing (Fig. 4C and fig. S8B).

This classification prompted us to investigate the contribution of each gene category to the rhythmicity of biological pathways. Clock-driven genes were enriched in circadian rhythm, while metabolic pathways were broadly enriched across all four categories (Fig. 4D and fig. S8). Pathways such as *N*-glycan biosynthesis showed stronger enrichment among mTOR-driven genes. Nonetheless, most pathways included a mixture of mTOR- and clock-driven rhythmic genes (Fig. 4D and fig. S8), reinforcing the idea that rhythmic biological processes are regulated by both circadian and mTOR signals. Representative genes from some key pathways are shown in figs. S3 and S4.

Together, our data demonstrate that inhibition of mTOR at the onset of its activity peak dissociates the phase of expression between clock- and mTOR-driven genes. Given that most biological pathways comprise genes whose rhythmicity depends on either the circadian clock or mTOR, perturbations to mTOR rhythmicity are likely to disrupt the temporal coordination of biological pathways.

### Clock- and mTOR-driven rhythmic genes are controlled by different TFs

**Fig. 5.**
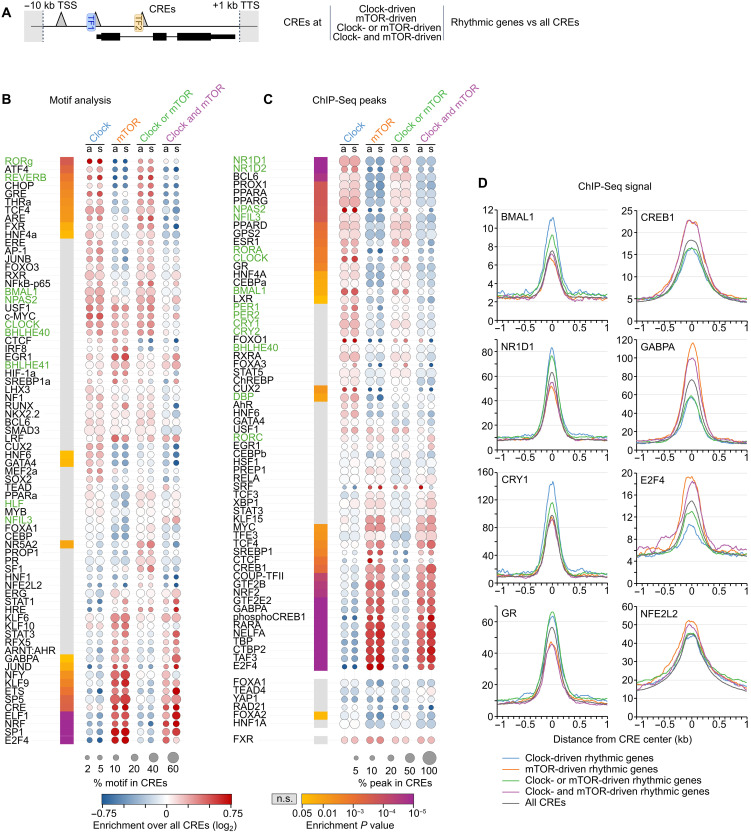
Clock- and mTOR-driven rhythmic genes are regulated by distinct transcriptional programs. (**A**) Schematic describing the strategy used for the motif and ChIP-seq peak enrichment analysis. TSS, transcription start site; TTS, transcription termination site. (**B**) TF motif enrichment at clock-driven, mTOR-driven, clock- or mTOR-driven, and clock- and mTOR-driven rhythmic genes. The red color indicates higher enrichment over all CREs (log_2_ scale), and the size of the circle represents the percentage of motifs found in CREs located in each of the four groups. Motifs with *P* ≤ 0.05 were considered significant. s, stringent list; a, complete list. (**C**) ChIP-seq peak enrichment at clock-driven, mTOR-driven, clock- or mTOR-driven, and clock- and mTOR-driven rhythmic genes. The red color indicates higher enrichment over all CREs (log_2_ scale), and the size of the circle represents the percentage of ChIP-seq peaks found in CREs located in each of the four groups. Motifs with *P* ≤ 0.05 were considered significant. s, stringent list; a, complete list. n.s., nonspecific. (**D**) Average ChIP-seq signal for eight TFs at CREs located in clock-driven (blue), mTOR-driven (orange), clock- or mTOR-driven (green), and clock- and mTOR-driven (purple) rhythmic genes. The average signal at all CREs is shown for comparison in gray.

Our finding that the phase of mTOR-driven genes can be dissociated from that of clock-driven genes (Fig. 4C) strongly suggests that these gene sets are regulated by distinct transcriptional programs, that is, mTOR-driven genes are likely controlled by TFs other than core circadian TFs. To test this possibility, we leveraged the nearly exclusive binding of TFs to open chromatin regions [cis-regulatory elements (CREs)] (*44*, *45*) and performed a motif enrichment analysis at CREs located within the loci of cycling genes from the four defined gene sets (Fig. 5A and table S6). This analysis confirmed that TF motifs were differentially enriched between groups. As expected, the RORE motif, bound by the circadian TFs REV-ERBs and RORs, was preferentially enriched in the clock-driven and clock- or mTOR-driven gene sets. The E-box motif, which is targeted by CLOCK, BMAL1, and NPAS2, was not significantly enriched in the clock-driven gene group, potentially due to widespread recognition of this motif by other basic helix-loop-helix TFs (see below). Conversely, motifs for TFs including E2F4, NRF1/2, CREB, SP1, KLF9, and members of the ETS (E26 transformation–specific) family, which regulate biological processes like stress response, detoxification, mitochondrial function, cell growth, and cell cycle, were preferentially enriched in the mTOR-driven and clock- and mTOR-driven gene groups (Fig. 5B).

To validate these findings, we reanalyzed public mouse liver chromatin immunoprecipitation sequencing (ChIP-seq) datasets for more than 60 TFs (table S7). Consistent with our motif enrichment results, ChIP-seq peaks for the circadian TFs NR1D1/2, RORA, BMAL1, CLOCK, and NPAS2 were enriched in the clock-driven and clock- or mTOR-driven gene groups (Fig. 5C). Conversely, peaks for TFs such as E2F4, CREB1, RARA, CTBP2, and GABPA were significantly enriched in mTOR-driven and clock- and mTOR-driven gene sets (Fig. 5C). ChIP-seq peaks for MYC, at TF that preferentially binds the canonical E-Box motif CACGTG also recognized by CLOCK:BMAL1, were enriched in the mTOR-driven and clock- and mTOR-driven gene groups. This suggests that TF binding to E-box motifs may be influenced by additional context-dependent mechanisms beyond the motif sequence alone (Fig. 5C) (*46*, *47*). Further examination of TF ChIP-seq signals for selected TFs including BMAL1, CREB1, GABPA, and GR supported the results of both the motif and peak enrichment analyses (Fig. 5D).

Together, our findings demonstrate that mTOR- and clock-driven rhythmic genes are targeted by distinct sets of TFs, supporting the idea that differential TF activity underlies the differences in rhythmic gene expression mediated by mTOR and the circadian clock.

### mTOR signaling contributes to the rhythmicity of the protein processing in the ER pathway and ER stress response

**Fig. 6.**
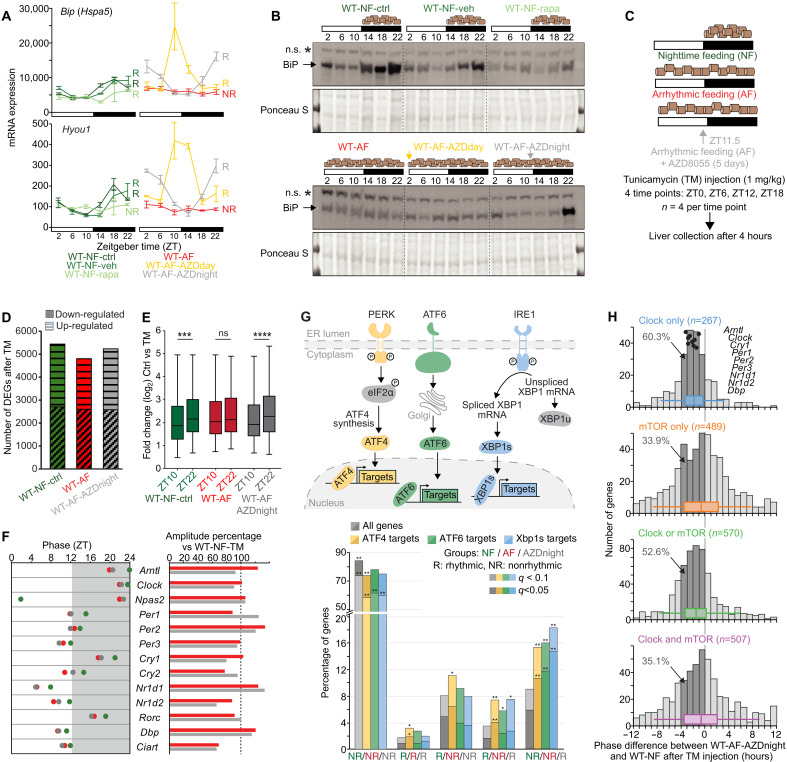
mTOR signaling regulates protein processing in the ER pathway and ER stress response. (**A**) Expression profiles of two ER chaperones in WT-NF-ctrl, WT-NF-veh, WT-NF-rapa, WT-AF, WT-AF-AZDday, and WT-AF-AZDnight mouse liver. Values represent the means ± SEM; *n* = 3 per time point. (**B**) Western blot analysis of BiP in the mouse liver. Ponceau stains of the same membranes are displayed below BiP blots. The asterisk denotes a nonspecific (n.s.) band. (**C**) Experimental design illustrating the different groups of mice, feeding regimens, and TM treatment schedule. (**D**) Number of DEGs (fold change superior or inferior to 1.5 and *P* ≤ 0.05) between TM-injected and control mice for each group (*n* = 18 for control mice; *n* = 16 for TM-injected mice). (**E**) Change in gene expression in response to TM injection for each group at ZT10 and ZT22. ***P < 0.001; ****P < 0.0001; ns: not significant. (**F**) Phase and amplitude (plotted as % of amplitude versus WT-NF-TM mice) of clock genes across different groups after TM injection. (**G**) Top: Schematic of the unfolded protein response pathway. Bottom: Enrichment of rhythmicity patterns among ER stress pathway targets. All genes and ATF4, ATF6, and XBP1s target genes were assigned to different rhythmicity categories on the basis of their rhythmicity pattern in WT-NF, WT-AF, and WT-AF-AZDnight after ER stress induction. Bars show the fraction of genes in each category; shaded portions indicate genes with *P* < 0.05 (light) or *q* < 0.05 (dark) rhythmicity thresholds. Asterisks denote categories in which branch-specific targets are significantly enriched relative to all expressed genes (Fisher’s exact test, *P < 0.05; **P < 0.01). (**H**) Phase difference between WT-AF-AZDnight and WT-NF-ctrl mice (gray) after TM injection for clock-driven, mTOR-driven, clock- or mTOR-driven, and clock- and mTOR-driven rhythmic genes. Negative values represent a phase advance in AZD8055-treated mice, while positive values represent a phase delay.

Our finding that rhythmic gene expression can be driven by either the circadian clock or mTOR suggests that alterations in mTOR activity may affect the rhythmicity of biological processes even when core clock oscillations remain intact. To test this possibility, we focused on the pathway “protein processing in the ER,” which we and others have found to be strongly regulated by mTOR (Figs. 1H and 4D and fig. S8D) (*48*, *49*) and is known to be regulated by food intake (*50*–*52*). This pathway involves ER chaperones that help with the proper folding and assembly of proteins in the ER lumen to maintain ER homeostasis and ensure protein quality control. The main ER chaperone *Hspa5* (also known as *BiP*/*Grp78*), along with the chaperones *Hyou1* and *Erp29*, exhibited rhythmic expression patterns in response to food intake rhythms and mTOR activity. Expression levels peaked in the second half of the night in night-fed mice, were dampened and/or arrhythmic in arrhythmically fed or rapamycin-treated mice, and were restored following AZD8055 injections (Fig. 6, A and B, and fig. S10, A to C).

ER chaperones also play a critical role during ER stress, a condition that arises when the ER is overwhelmed with misfolded or unfolded proteins and that leads to the activation of the unfolded protein response (UPR) pathway (*53*, *54*). While initially protective, persistence of ER stress ultimately leads to cell death. The ER stress response has been reported to be rhythmic with a period of 12 hours (*55*, *56*) and induced by different feeding regimens (*50*, *51*, *56*–*58*). Moreover, accumulating data indicate that the mTOR signaling pathway interacts with UPR response pathways in the liver (*48*, *52*, *59*, *60*), raising the possibility that mTOR contributes to the temporal regulation of ER stress. This and our observation that ER chaperone expression peaked in the second half of the night in response to food intake and mTOR signaling (Fig. 6, A and B) prompted us to test whether the rhythmicity of the ER stress response depends on rhythmic mTOR activity. To that end, we induced ER stress by injecting tunicamycin (TM) at four time points (ZT0, ZT6, ZT12, and ZT18) in three groups of WT mice: WT-NF, WT-AF, and WT-AF-AZDnight. Livers from these mice were then harvested 4 hours after TM injection at ZT4, ZT10, ZT16, and ZT22 (Fig. 6C). A comparison of liver gene expression between TM-injected mice (after combining all samples and time points; *n* = 16 per group) and corresponding uninduced control mice (after combining all samples and time points; *n* = 18 per group) confirmed robust ER stress induction. About 30% of differentially expressed genes (DEGs) were identified across all three groups, indicating that TM induction of ER stress was effective and group-independent (Fig. 6F, fig. S10D, and tables S8 and S9). As expected, DEGs were strongly enriched in protein processing in the ER pathway for each group (fig. S10E).

To determine whether the ER stress response rhythm is regulated by mTOR signaling, we compared gene expression between uninduced and TM-treated mice for each group at ZT10 and ZT22, i.e., the two time points available for both uninduced and TM-treated mice. Consistent with the reported time-of-day difference in ER stress response (*56*), TM induced larger changes in gene expression at ZT22 than at ZT10 in WT-NF mice (Fig. 6E). This time-of-day difference was blunted in arrhythmically fed mice but restored by AZD8055 treatment (Fig. 6E). Given that core clock gene rhythmic expression remained similar across all TM-treated groups (Fig. 6F and fig. S11), these results indicate that the ER stress response is a rhythmic process that primarily relies on RFI and rhythmic mTOR activity rather than on circadian clock oscillations, in line with previous findings (*55*).

The UPR transcriptional response is mediated via three independent pathways: the ATF6, IRE1/XBP1, and PERK/ATF4 signaling branches, each of which activates distinct sets of target genes to restore ER homeostasis (Fig. 6G) (*53*, *54*). We analyzed published ATF4, ATF6, and XBP1s target gene sets (*60*–*62*) and classified these genes into distinct categories according to their rhythmicity across WT-NF, WT-AF, and WT-AF-AZDnight conditions after ER stress induction. Compared to all expressed genes, the three UPR branches were significantly enriched for genes that were nonrhythmic under AF but regained rhythmicity upon AZD8055 treatment (Fig. 6G), highlighting a global regulatory role of rhythmic mTOR activity in the hepatic ER stress response. Analysis of peak expression times for UPR target genes (fig. S12A) and markers (fig. S12B) revealed that although AZD8055 treatment rescued rhythmicity under AF treatment, it did not delay the induction of ER stress response genes, i.e., peak expression clustered around dawn in both control and AZD8055-treated mice (ZT2 and ZT22, respectively; fig. S12C). This contrasts with the global phase delay observed for mTOR-driven genes in untreated WT-AF-AZDnight mice (Fig. 4C). To reconcile this difference, we compared expression phases between WT-AF-AZDnight and WT-NF mice after TM treatment across the four gene categories. Clock-driven genes, including all core clock components, and clock- or mTOR-driven genes exhibited an average phase advance of ∼2 hours in WT-AF-AZDnight mice (Fig. 6H and fig. S12D). In contrast, mTOR-driven genes (and, to a lesser extent, clock- and mTOR-driven genes) displayed more modest phase advances, and phase relationships were considerably more heterogeneous. After TM treatment, only 33.9% of genes in WT-AF-AZDnight mice fell within a 0- to 4-hour advance window relative to WT-NF compared with 60.3% for clock-driven genes. This greater dispersion may reflect differences in how ER stress–activated TFs (XBP1s, ATF4, and ATF6) are integrated into the transcriptional networks that confer mTOR-driven rhythmicity, as opposed to their more uniform interaction with circadian TFs (Fig. 5). In summary, our results suggest that protein processing in the ER pathway and the ER stress response are preferentially regulated by RFI and require rhythmic mTOR activity to elicit an efficient rhythmic response to ER stress.

### mTOR inhibition can rescue the rhythmicity of metabolites that is lost upon AF

**Fig. 7.**
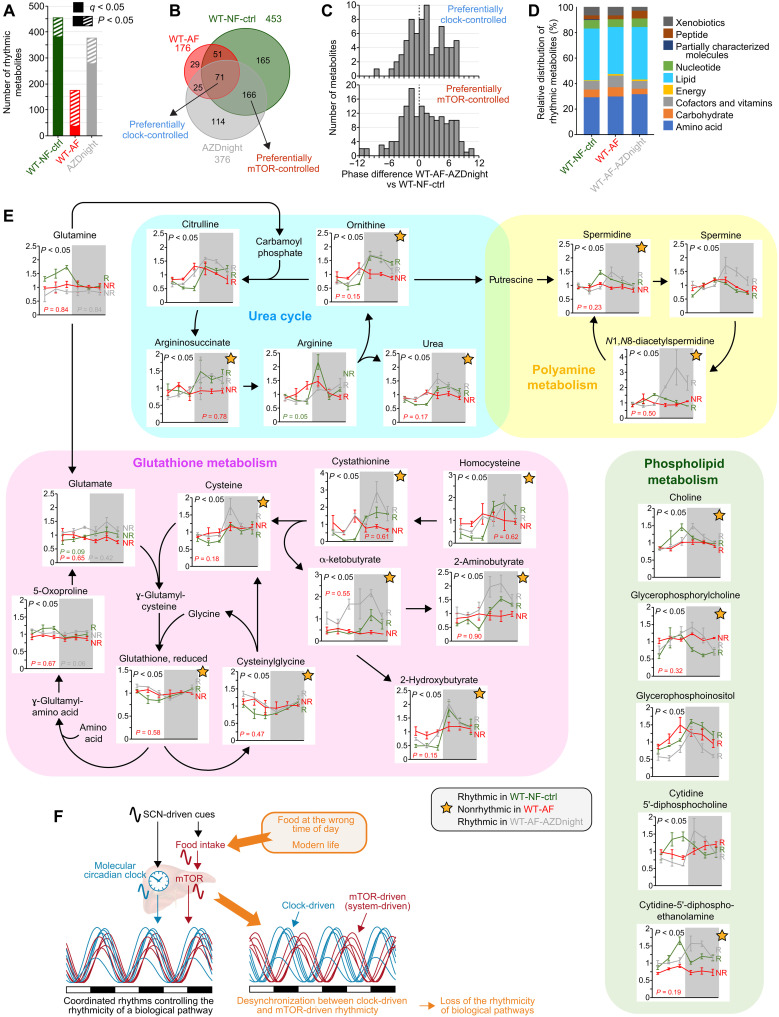
mTOR rhythmic activity contributes to the mouse liver cycling metabolome. (**A**) Number of rhythmic metabolites detected in each group of mice (WT-NF-ctrl, WT-AF, and WT-AF-AZDnight). (**B**) Overlap of rhythmic metabolites. (**C**) Phase difference between WT-NF-ctrl and WT-AF-AZDnight mice (gray) for clock- and mTOR-driven rhythmic metabolites. Negative values represent a phase advance in AZD8055-treated mice, while positive values represent a phase delay. (**D**) Class distribution of rhythmic metabolites in WT-NF-ctrl, WT-AF, and WT-AF-AZDnight mice. (**E**) Profiles of various metabolites in WT-NF-ctrl, WT-AF, and WT-AF-AZDnight mice. (**F**) Proposed model for clock- and mTOR-controlled rhythms in the mouse liver.

To extend our findings beyond gene expression, we performed untargeted global metabolomic profiling in the liver across the 24-hour day. This analysis revealed that AF severely reduced the number of rhythmic metabolites compared to WT-NF-ctrl mice and that acute mTOR inhibition at ZT11.5 largely rescued their rhythmicity (Fig. 7A and tables S10 and S11). Overlap analysis identified 166 metabolites whose rhythmicity was dependent on rhythmic mTOR activity, that is, they were rhythmic in NF, lost rhythmicity in AF, and were rescued by acute mTOR inhibition (Fig. 7B). A phase comparison between WT-NF-ctrl and WT-AF-AZDnight mice showed no differences between mTOR- and clock-driven rhythmic metabolites, i.e., both groups displayed a comparable wide range of phase differences (Fig. 7C). This contrasts with the phase differences observed at the gene expression level and may be explained by the flux of metabolites within metabolic pathways that constrains metabolite levels across a large number of metabolic reactions. Notably, the class distribution of rhythmic metabolites was comparable between groups, indicating that RFI and mTOR activity globally influence metabolism rather than acting on specific classes of metabolites (Fig. 7D). This result is consistent with our finding that most biological pathways include both clock- and mTOR-driven rhythmic genes (Figs. 1H and 4D).

Many metabolites whose rhythmicity was preferentially driven by mTOR activity were involved in the urea cycle, glutathione metabolism, polyamine metabolism, and phospholipid metabolism (Fig. 7E). For instance, several metabolites within the glutathione pathway like homocysteine, cystathionine, cysteine, α-ketobutyrate, 2-aminobutyrate, 2-hydroxybutyrate, cysteinylglycine, and reduced glutathione were rhythmic under NF, lost rhythmicity under AF treatment, and were restored by acute mTOR inhibition. Prior work has shown that polyamine biosynthesis is regulated by circadian and feeding cues (Fig. 7E) (*63*, *64*). Supporting this, we found that expression of *Srm*, the gene encoding spermidine synthase that converts putrescine to spermidine, was rhythmic in NF mice, dampened under AF and rapamycin treatment, and rescued by acute mTOR inhibition (fig. S13A). Consistent with *Srm* expression, the polyamines spermidine and *N*^1^,*N*^8^-diacetylspermidine also showed mTOR-driven rhythmicity (putrescine levels were below the detection threshold). Additional examples of enzymes within the urea cycle and glutathione metabolism pathways, whose rhythmic expression is regulated in part by mTOR activity and/or the clock, are provided in fig. S13 (B and C).

Together, these findings reveal that the rhythmicity of most liver metabolites depends on RFI and rhythmic mTOR activity. This underscores the broad influence of mTOR on gene expression and metabolism, with important implications for cellular homeostasis and organismal physiology.

## DISCUSSION

Despite mounting evidence that RFI initiates system-driven rhythms in gene expression independently of the circadian clock (*18*–*21*), the underlying mechanism has remained elusive. Using various pharmacological approaches and feeding paradigms, we demonstrate that the rhythmic activity of the nutrient-sensing kinase mTOR is both necessary and sufficient to drive rhythms in gene expression in the mouse liver. Inhibition of rhythmic mTOR activity prevents the rhythmic expression of hundreds of genes, whereas its resetting restores the cycling of hundreds of genes, all of this without affecting core clock gene oscillations. Acute inhibition of mTOR at dusk in AF mice delays the expression of mTOR-driven rhythmic genes but not clock-driven rhythmic genes. Given that most rhythmic biological processes include both clock- and mTOR-driven rhythmic genes, this strongly suggests that misalignment between clock- and mTOR-driven signals can desynchronize the coordinated rhythms in gene expression and, in turn, compromise the rhythmicity of biological processes.

Connections between the molecular circadian clock and mTOR have been reported (*26*, *65*–*70*). In particular, S6K1-mediated phosphorylation of BMAL1 has been shown to facilitate BMAL1-dependent stimulation of protein synthesis through association with the translational machinery in the cytosol. However, our data indicate that the loss of BMAL1 does not prevent rapamycin-induced suppression of S6K phosphorylation and decrease in food-driven rhythms in gene expression. This suggests that while mTOR and the core clock clearly interact, mTOR does not strictly require an intact core clock to affect downstream rhythmic processes, including cycling transcription.

Previous reports showing that mTOR senses nutrient availability to promote anabolic processes (*22*, *23*) and that mTOR activity oscillates across the 24-hour day in response to food intake (*19*, *24*–*28*) strongly support our findings that mTOR contributes to the hepatic cycling transcriptome. However, finding that mTOR inhibition profoundly disrupts rhythmic gene expression without affecting the circadian clock is more unexpected given reports showing that mTOR signaling can regulate the circadian clock function (*69*, *71*, *72*). Yet, the most potent effects in mammals were observed in cell culture or ex vivo, and in vivo effects were more modest and restricted to circadian period defects (*69*) or following mTOR deletion in *Vip* neurons (a key SCN neuronal subpopulation), which may have led to cellular complications given that constitutive mTOR knockout is embryonically lethal (*73*–*75*). We propose that the lack of robust effects of mTOR inhibition on core clock oscillations in vivo in the mouse liver may reflect the redundant signals that the liver clock experiences and that maintain clock function (e.g., daily rhythms of body temperature, neuronal signals, hormones, etc.) and are absent in in vitro and ex vivo systems, rendering the clock more susceptible to mTOR manipulation.

Although short-term mTOR inhibition by rapamycin specifically inhibits mTORC1, prolonged rapamycin treatment can also alter mTORC2 assembly and inhibit its activity (*34*). It has also been shown that many rapamycin-regulated genes are mTORC2/Rictor–dependent (*76*). We found that pS473 AKT, a target of mTORC2 (*35*, *36*), does not exhibit robust and consistent rhythms (fig. S5), at least when compared to the mTORC1 target pS6K (Figs. 1B and 2E and figs. S2, A and B, and S6, C to E). Rapamycin treatment also affected more profoundly pS6K levels than pS473 AKT levels, especially in WT mice. Moreover, *Tsc1^LKO^* had limited effects on the cycling liver transcriptome despite blunting mTORC2 activity (Fig. 2B and fig. S5). This suggests that rhythmic mTOR outputs seem to be more closely associated with mTORC1 activity than with canonical mTORC2 signaling, although we cannot completely rule out the notion that chronic rapamycin treatment impairs mTORC2 function and that some rapamycin-responsive genes and genes categorized as mTOR-driven rhythmic genes may reflect combined mTORC1/mTORC2 regulation.

Nutrient inputs to mTOR include amino acids, insulin, and glucose, which signals via distinct regulators including *Raptor* (amino acid sensing) and *Tsc1* (insulin signaling) (Fig. 2A). Genetic deletion of *Raptor* and *Tsc1* in the mouse liver had limited effects on the hepatic cycling transcriptome, likely because input pathways redundantly regulate rhythmic mTOR activity. This is supported by the elevated yet rhythmic levels of phospho-S6K in *Tsc1^LKO^* mice (Fig. 2C). This also suggests that targeting specific macronutrient pathways may not be sufficient to manipulate rhythmic mTOR activity, i.e., mTOR activity is likely to only be sensitive to global changes of energy balance rather than temporal changes of specific nutrient inputs.

The different effects of rapamycin on the amplitude and AZD8055 on the phase are not contradictory but instead reflect differences in their mechanisms of action and in the experimental context. In NF mice, where mTOR activity is already rhythmic, rapamycin acts as a long-lasting inhibitor of mTORC1 because of its long half-life (*39*). This leads to a reduction of peak mTOR activity and decreased amplitude without generating a sharp on-off transition that would be required to reset the phase. In contrast, in AF mice, AZD8055 effectively resets the phase of mTOR-driven gene expression to the time of injection. This is consistent with the properties of ATP-competitive mTOR inhibitors, which produce stronger and more uniform inhibition of mTOR signaling (*77*, *78*). AZD8055 inhibition is transient because of rapid drug clearance, allowing mTOR signaling to recover after a few hours. This recovery enables mTOR signaling to realign with the timing of inhibition, resulting in an ∼12-hour phase difference between AZD-day and AZD-night groups (Fig. 4C).

Characterization of cycling transcriptomes across mammalian tissues revealed that about half of the genome is rhythmically expressed somewhere in the body (*1*–*4*). Our finding that mTOR inhibition at dusk in AF mice delays the phase of mTOR-driven genes without affecting the phase of clock-driven genes indicates that not all rhythmic genes are regulated by the canonical circadian TFs (CLOCK:BMAL1, REV-ERBs, and RORs) or their direct downstream targets (e.g., DBP, TEF, HLF, NFIL3, DEC1, and DEC2). This notion is supported by our TF motif enrichment and ChIP-seq analyses, which revealed that mTOR-driven rhythmic genes are preferentially targeted by a different set of TFs that include E2F4, CREB1, CTBP2, GABPA, NRF2, TFE3, and SREBP1 (Fig. 5C). Notably, these TFs are themselves directly regulated by mTOR or by the cellular metabolic state. TFE3 transcriptional activity is inhibited by mTOR-mediated phosphorylation (*79*, *80*), SREBP1 is indirectly activated by mTORC1 via phosphorylation of Lipin1 (*81*), and CREB1 activity is regulated by phosphorylation from S6K, which is itself activated by mTORC1 (*82*). Moreover, CTBP2 repressor activity is regulated by NADH [reduced form of nicotinamide adenine dinucleotide (oxidized form)] and acyl-coenzyme A (*83*, *84*), GABPA is activated by extracellular signal–regulated kinase 2–mediated phosphorylation and redox state (*85*, *86*) to regulate the mitochondrial function and energy metabolism (*87*), and NRF2 is activated upon oxidative and electrophilic stresses (*88*) to promote antioxidant response and cellular detoxification (*87*). Together, these data therefore support the notion that daily changes in nutrient availability, energy balance, and redox state regulate the activity of some specific TFs including via mTOR to, in turn, drive diurnal rhythms in gene expression independently of the circadian clock.

Under physiological conditions where system-level rhythms are aligned to the circadian cycle, all rhythmic signals likely act in concert to coordinate TF activity and promote synchronized oscillations in gene expression that support the rhythmicity of biological processes. However, and as seen with acute mTOR inhibition at dusk (Fig. 4C), misalignment between system- and clock-driven signals is likely to disrupt the phase relationship between circadian TFs and mTOR/system–driven TFs and consequently disrupt the phase relationship between all rhythmic transcripts (Fig. 7F). Given that mTOR- and clock-driven rhythmic genes converge onto similar pathways (Fig. 4D), this phase misalignment may compromise pathway-level coherence and ultimately impair rhythmic biological functions, potentially manifesting as CRD (Fig. 7F). Future studies will be required to directly test this model.

Our investigation of the ER stress response pathway offers partial support. Although our temporal resolution does not allow direct observation of the 12-hour rhythm of endogenous ER stress activation (*55*, *56*), we were able to detect time-of-day differences in the ER stress response, consistent with previous reports (*55*, *56*, *89*). Notably, nighttime AZD8055 treatment in AF mice elicited a pronounced ER stress response at ZT22 (Fig. 6 and figs. S10 and S12), revealing a temporally restricted window of activation. This notable temporal specificity raises the possibility that acute resetting of mTOR activity not only restores rhythmicity but also creates a defined window of heightened ER stress responsiveness. One potential explanation is a transient rebound in mTOR activity following AZD8055 clearance. Because AZD8055 is rapidly eliminated, inhibition at ZT11.5 may be followed by a recovery phase several hours later, resulting in a sharp increase in mTOR signaling that could transiently elevate protein synthesis and ER load independently of feeding rhythms. Alternatively, AZD8055 may restore the coordination between feeding-driven signals and ER chaperone expression, producing a temporally gated window in which the unfolded protein response is most robust. While these mechanisms remain to be tested, this temporally restricted response is consistent with a broader role for rhythmic mTOR activity in aligning hepatic stress-response pathways with systemic metabolic cues. The enrichment of mTOR rhythmicity–sensitive genes across all three branches of the UPR further supports this role.

Time-of-day differences in the ER stress response are in line with previous findings establishing the influence of feeding regimen on hepatic ER stress (*50*–*52*, *56*–*58*) and with the interplay between mTOR signaling and ER stress (*48*, *52*, *59*, *60*). At the same time, our results also suggest that intricate interactions between transcriptional programs can produce complex transcriptional outputs. For example, ER stress–induced TFs (i.e., ATF4, AF6, and XBP1s) increased phase heterogeneity and reduced the phase delays of mTOR-driven genes in WT-AF-AZDnight mice while simultaneously advancing uniformly the phase of clock-driven genes by ∼2 hours. These contrasting effects may reflect differential integration of ER stress–activated TFs with the gene networks governed by mTOR versus those governed by circadian TFs, potentially resulting in greater phase variability in the mTOR-driven network (fig. S13B).

Together, our transcriptomics and metabolomics analyses highlight the potential of targeting mTOR rhythmic activity as a strategy to restore rhythmic gene expression. Our findings are limited to the liver, and whether similar mechanisms operate in other tissues, including the brain, remains an open question. Nevertheless, given mTOR’s well-established role in regulating cell growth, metabolism, and survival, and its involvement in diseases like cancer, several pharmacological tools are already available and could be repurposed to therapeutically modulate rhythmic mTOR signaling.

In conclusion, our work reveals a central role for the nutrient-sensing kinase mTOR in driving daily rhythms of gene expression in the mouse liver, independently of the circadian clock machinery. Whether mTOR plays a similar role across other tissues remains an open question. Given its function as a primary nutrient sensor, it is plausible that mTOR fulfills a comparable role in tissues where nutrient availability predominantly shapes metabolic rhythmicity. It is also tempting to speculate that mTOR contribution to cycling transcriptomes is substituted by other signaling effectors in tissues being sensitive to other cues, e.g., the fact that rhythms in neuronal activity bypass the circadian clock to drive rhythmic gene expression in the brain. Overall, our findings uncover an additional mechanism that contributes to 24-hour transcriptional rhythms, offering a potential explanation for how environmental changes or physiological stressors can reprogram circadian transcriptional programs without substantially disrupting core clock oscillations (*90*–*94*). These results also suggest that strategies aimed at restoring robust, clock-aligned rhythms in mTOR activity could help alleviate some of the symptoms associated with CRD.

## MATERIALS AND METHODS

### Animals

Male C57BL/6NCrl and *Bmal1^−/−^* mice (ages ranging from 2 to 4 months old) were raised in-house on a 12-hour light:12-hour dark cycle (LD 12:12). *Tsc1* floxed mutant mice (*Tsc1tm1Djk/J*; the Jackson Laboratory, stock no. 005680), *Rptor* floxed mutant mice (B6.Cg-*Rptor^tm1.1Dmsa^*/J; the Jackson Laboratory, stock no. 013188), and *Alb-Cre* mice [B6.Cg-Speer6-ps1Tg(Alb-cre)21Mgn/J; the Jackson Laboratory, stock no. 003574] were purchased from the Jackson Laboratory and raised in-house on a 12-hour light:12-hour dark cycle (LD 12:12). *Alb-cre* mice were crossed with *Tsc1^flox^* or *Rptor^flox^* mice to generate liver-specific *Tsc1* knockout (*Tsc1^LKO^*) or *Rptor* knockout (*Rptor^LKO^*) mice. *Alb-cre* mice were used as control. At the start of the experiment, mice were housed in individual cages with ad libitum access to food (45 mg of dustless precision pellets, grain-based; F0165; Bio-Serv) and water. Animals were then assigned to feeding groups with respective vehicle or drug treatment groups (*n* = 18 mice per group). All animals were used in accordance with the guidelines set forth by the Institutional Animal Care and Use Committee of Texas A&M University (AUP no. 2022-0050).

### Feeding system

The feeding system used in our study was described previously (*19*, *29*) and consists of an eight-compartment clear plastic organizer placed on top of a 24-hour timer. The food container is covered with a lid such that mice have access to one compartment every 3 hours. The timers are tested for effective rotations before introducing the mice individually in the cages.

### Rhythmic food intake manipulation

All mice were fed with 45 mg of dustless precision pellets, grain-based (F0165, Bio-Serv) composed of protein (21.3%), fat (3.8%), fibers (4%), ash (8.1%), carbohydrates (54%), and moisture (<10%). One gram of food pellet was equivalent to 3.35 kcal. All mice were first acclimated to the 12-hour (LD 12:12) light:dark cycle with ad libitum access to food and water without using the feeding system for 1 week. They were then weighed and assigned to individual cages under different experimental conditions. For the following 3 to 7 days, mice were given access to ad libitum food (1.5 g per compartment) to acclimate them to the feeding system. The amount of food given and consumed by each mouse was calculated daily to collect a baseline feeding profile. Mice were then gradually transitioned from ad libitum to either NF or AF depending on their respective feeding regimens by gradually reducing the amount of food in the daytime compartments for NF mice and from all eight compartments for AF mice. AF was achieved by splitting the average daily food consumption evenly between the eight food compartments, while NF was achieved by splitting the average daily food consumption evenly between the four compartments exposed at night. All mice had ad libitum access to water. Food changeouts were done at ZT8 (3 p.m.) every day. The food given and food consumed per mouse were noted daily to keep track of the amount of food consumed every 3 hours. Mice were subjected to their feeding regimens for at least 2.5 weeks before they were euthanized and tissues were collected.

### Pharmacological inhibition of mTOR with rapamycin and AZD8055

Mice fed only at night for 2 weeks were injected with rapamycin intraperitoneally at 8 mg/kg every other day for 2 weeks. Mice were euthanized 18 hours (ZT02) to 38 hours (ZT22) after the last rapamycin injection. Rapamycin stock was made by resuspending it in ethanol (20 mg/ml). Rapamycin was mixed with the vehicle (0.25% Tween 80 and 0.25% polyethylene glycol, molecular weight 400: final concentration of 1.2 mg/ml) just before injection. Vehicle-injected mice were injected with vehicle and ethanol (same amount as that of rapamycin used for drug-treated mice). Injections were carried out at ZT8 and ZT9 just after food changeouts to avoid disturbing the mice multiple times a day.

For treatment with the short half-life mTOR inhibitor AZD8055, mice under AF schedule for 2 weeks were intraperitoneally injected with AD8055 at 10 mg/kg intraperitoneally for five consecutive days. One group of mice (*n* = 18) received AZD8055 at ZT0, just at the beginning of the light phase, and the other group of mice (*n* = 18) received AZD8055 at ZT11.5, just before the onset of the dark phase. A 5× AZD8055 stock was made in 30% Captisol and diluted to 1× with 30% Captisol just before injection.

### RNA extraction and processing

Mice were anaesthetized with isoflurane and decapitated, and the liver was collected, briefly rinsed in ice-cold 1× phosphate-buffered saline, flash-frozen in liquid nitrogen, and stored at −80°C. We used three equal-sized pieces of the left lateral lobe: one for RNA extraction, one for protein, and one for metabolome analysis. RNA was extracted using TRIzol reagent following the manufacturer’s instructions (Life Technologies). Briefly, 300 μl of TRIzol was added to the frozen tissue, and the tissue was then homogenized using an electric hand-held pellet mixer. The volume was then brought to 1 ml by adding 700 μl of TRIzol. Two hundred microliters of chloroform was added, mixed well, and centrifuged at 12,000*g* for 15 min at 4°C. The aqueous phase was isolated, mixed with 1 volume of isopropanol, and incubated for an hour in −20°C. Tubes were then centrifuged at 12,000*g* for 10 min at 4°C, and the RNA pellet was washed with 75% ethanol and lastly resuspended in deionized water. Total RNA was further purified with the acid phenol/chloroform extraction method and precipitated using ethanol. Total RNA was quantified using a NanoDrop-1000. The quality and integrity of RNA were verified by running the RNA on a 1.5% agarose gel.

### Library preparation and 3′ mRNA sequencing

RNA sequencing (RNA-seq) libraries were generated using the Lexogen QuantSeq 3′mRNA-Seq Library Prep Kit following the manufacturer’s instructions. Two micrograms of total RNA was used as the starting material for first-strand synthesis. Following RNA removal, second-strand synthesis, and purification, the libraries were amplified by polymerase chain reaction for 12 cycles according to the manufacturer’s recommendations for mouse liver tissue. Libraries were multiplexed using the i7 indices and then mixed in equimolar concentrations. The libraries were sequenced using an Illumina NextSeq 500 platform with a sequencing length of 75 nucleotides (nt). For ER stress induction experiments and *Rptor^LKO^* mice, the parameters for library preparation and sequencing were slightly different with 0.5 μg of total RNA used, and sequencing was performed using an Illumina NextSeq 2000 platform with a sequencing length of 75 nt for ER stress induction experiments and 100 nt for *Rptor^LKO^* mice.

### Sequencing data processing

Sequenced reads were preprocessed using the R package ShortRead (*95*) to remove the first 12 nt; remove low-quality bases at the 3′ end; for polyadenylate tail trimming; and to remove all reads shorter than 36 nt in length. Reads were then aligned to the mm10 transcriptome, assembly GRCm38.p6, Gencode release M25, using the STAR aligner (*96*). Secondary alignments were removed with samtools view -F 0X100. Read counts were summarized with the summarizeOverlaps function from the R package GenomicRanges (*97*) using the options “mode = IntersectionStrict and inter.feature = FALSE” and normalized by using a between-sample normalization method using the dryR package (*21*). The final count matrix contains 15,907 genes (table S1).

### Rhythmicity analysis

Rhythmicity analysis was performed using four algorithms: F24 (*98*), JTK_CYCLE (from MetaCycle) (*99*), RAIN (*100*), and HarmonicRegression (*101*). The four resulting *P* values were combined using a harmonic mean *P* value (hmp) method and adjusted to control for the false discovery rate using the Benjamini-Hochberg (BH) method using the p.adjust function in baseR. The outcome of the rhythmicity analysis is provided in table S2 and fig. S1A. Genes with a BH-adjusted *P* (*q*) ≤ 0.05 were considered as rhythmic, except for the characterization of clock- and mTOR-driven rhythmic genes (see below). The relative amplitude (rAMP) reported by MetaCycle was used for amplitude analysis, whereas the phase given by HarmonicRegression was used for phase analysis. Fold-change expression was calculated by dividing the maximal expression (average of the three biological replicates) to the minimal expression (average of the three biological replicates). Values for these parameters are provided in table S3.

### Characterization of clock- and mTOR-driven rhythmic genes

To identity genes whose rhythmicity is controlled by the circadian clock and/or mTOR, we sought to compare rhythmic genes between the nine following groups: WT-NF-ctrl, WT-NF-veh, WT-NF-rapa, WT-AF, BMKO-NF-ctrl, BMKO-NF-veh, AC-NF, WT-AF-AZDday, and WT-AF-AZDnight. *Tsc1^LKO^* and *Rptor^LKO^* mice were not included because they exhibited a large number of rhythmic genes, yet we could not exclude the notion that the rhythmic expression of some mTOR-driven genes was impaired. Given that no algorithm can simultaneously perform a differential analysis of rhythmicity between nine experimental conditions, we opted for a more conventional analysis relying on the comparison of *P* values. To that end, we proceeded to the following stepwise procedure:

1) We first combined the harmonic mean of the *P* values obtained from the four rhythmicity algorithms for the three control groups WT-NF-ctrl, WT-NF-veh, and AC-NF-ctrl (see above) using the hmp method to obtain a single *P* value for each gene for nighttime-fed WT/control mice, where both clock and mTOR signals are rhythmic (group called WT-NF-ave hereafter).

2) Similarly, we combined the hmp for BMKO-NF-ctrl and BMKO-NF-veh to obtain a single *P* value per gene for nighttime-fed *Bmal1^−/−^* mice, where mTOR activity is rhythmic and the circadian clock output is arrhythmic (group called BMKO-NF-ave hereafter).

3) We then considered that clock-driven rhythmic genes should be rhythmic (hmp ≤ 0.05) in WT-NF-ave, WT-NF-rapa, WT-AF-ctrl, WT-AF-AZDday, and WT-AF-AZDnight but nonrhythmic (hmp > 0.1) in BMKO-NF-ave. We chose hmp > 0.1 for nonrhythmic genes to avoid including genes having an hmp close to the significance threshold.

4) Similarly, we considered that mTOR-driven rhythmic genes should be rhythmic (hmp ≤ 0.05) in WT-NF-ave, BMKO-NF-ave, WT-AF-AZDday, and WT-AF-AZDnight but nonrhythmic (hmp > 0.1) in WT-NF-rapa and WT-AR-Ctrl.

5) Genes rhythmic (hmp ≤ 0.05) in all six conditions (WT-NF-ave, BMKO-NF-ave, WT-NF-rapa, WT-AF, WT-AF-AZDday, and WT-AF-AZDnight) were labeled as clock- or mTOR-driven, i.e., they remained rhythmically expressed even if one of the two rhythmic input is lost.

6) Genes rhythmic (hmp ≤ 0.05) in all WT-NF-ave, WT-AF-AZDday, and WT-AF-AZDnight but nonrhythmic (hmp > 0.1) in BMKO-NF-ave, WT-NF-rapa, and WT-AF were labeled as clock- and mTOR-driven, i.e., their rhythmic expression required both clock and mTOR inputs to be rhythmic.

7) This analysis led to the identification of 169 clock-driven, 256 mTOR-driven, 354 clock- or mTOR-driven, and 159 clock- and mTOR-driven rhythmic genes.

8) Because this analysis is stringent and requires significant *P* values in 6+ independent rhythms, we considered loosening the cutoffs to obtain a more complete list of genes. We reasoned that a gene with hmp ≤ 0.1 in three or more independent experimental conditions is more likely to be rhythmic than nonrhythmic. Thus, we repeated steps 3 to 6 and called a gene rhythmic if hmp ≤ 0.1 between the relevant groups and nonrhythmic if hmp > 0.05. This analysis led to the identification of 267 clock-driven, 489 mTOR-driven, 570 clock- or mTOR-driven, and 507 clock- and mTOR-driven rhythmic genes.

Downstream analysis (gene expression profile, phase, motif enrichment, TF ChIP-seq peaks, etc.) between the different groups of rhythmic genes did not reveal differences between the stringent and more complete list of genes such that we described findings for both lists here. The full list of genes is provided in table S5.

### Computation of average rhythmic expression

The average rhythmic expression was computed to display differences in rhythmicity profiles between groups, as in Fig. 4A. First, the RNA-seq signal of each rhythmic gene was normalized to the average signal of the 18 samples of each rhythm (six time points with *n* = 3 per time point). Resulting mean-normalized signals were then averaged on the basis of the phase of expression of WT-NF-ctrl mice and by bin of 4 hours, i.e., the mean-normalized RNA-seq signal was averaged for all genes having a peak of expression in WT-NF-ctrl mice from ZT0 to ZT4, from ZT4 to ZT8, from ZT8 to ZT12, etc. The resulting signals were then centered on the basis of the peak of expression for each replicate and for each bin and used to calculate the means +/− 95% confidence interval. In other words, values from the peak expression at ZT0 to ZT4, ZT4 to ZT8, ZT8 to ZT12, ZT12 to ZT16, ZT16 to ZT20, and ZT20 to ZT24 and for each rhythm replicate were used to calculate the means +/− 95% confidence interval. The same was done for the next 4-hour bins to provide a full average signal over 24 hours, representing all rhythmic genes within a group.

### KEGG enrichment analysis

KEGG enrichment analysis was performed using the kegga function available in the R package Limma (*102*).

### Motif analysis

Because TFs almost exclusively bind DNA at CREs (encompass enhancers and promoters) (*44*, *45*), motif analysis was restricted to CREs located within each gene locus. We first used a public list of mouse liver deoxyribonuclease I–hypersensitive sites, which was derived from deoxyribonuclease sequencing datasets from the ENCODE project and identified mouse liver CREs across the genome (*19*, *103*). Analysis was performed using the top 20,000 CREs. CREs located within 10 kb upstream the transcription start site to up to 1 kb downstream the transcription termination site of every gene of interest were kept, resulting in 602 deoxyribonuclease I hypersensitive sites for clock-driven, 751 for mTOR-driven, 1524 for clock- or mTOR-driven, and 766 for clock- and mTOR-driven groups (for the complete list of genes, see above). CRE fasta sequences for each CRE were retrieved and used with the findMotifs.pl script of the HOMER suite. Motif enrichment was calculated by comparing motif enrichment for each group to the background enrichment calculated at all top 20,000 CREs. Motifs were considered significantly enriched if *P* ≤ 0.05 (Fisher’s exact test). Raw data for the motif enrichment are provided in table S6.

### Analysis of public mouse liver TF ChIP-seq signal

To determine whether specific TFs are recruited to clock- and/or mTOR-driven genes, we first reanalyzed public mouse liver ChIP-seq datasets of more than 65 TFs (table S7). Raw fastq files were uploaded using the sra toolkit and mapped to the mm39 genome with either bowtie (reads ≤50 nt) or bowtie2 (reads >50 nt). ChIP-seq peaks were identified using MACS2 using standard parameters and set to the top 20,000 most enriched peaks for TFs exhibiting more than 20,000 ChIP-seq peaks. The overlap between TF ChIP-seq peaks and CREs at clock- and/or mTOR-driven genes was determined using the intersectBed function of Bedtools. Enrichment for TF ChIP-seq was calculated by comparing the number of TF ChIP-seq peaks for each group to the background enrichment calculated at all top 20,000 CREs. TF ChIP-seq peaks were considered significantly enriched if *P* ≤ 0.05 (Fisher’s exact test).

### Western blotting

Frozen liver tissue pieces (one piece of the left lateral lobe) were homogenized in 300 μl of ice-cold lysis buffer (20 mM Hepes, 50 mM KCl, 10% glycerol, 2 mM EDTA, 1% Triton X-100, 0.4% NP-40, 1× protease inhibitor cocktail, 1 mM dithiothreitol, and 1× phosphatase inhibitor cocktails I and III) using three cycles of 30-s homogenization on ice. The homogenate was centrifuged at high speed for 10 min at 4°C, and the supernatant was collected. The protein concentration was determined using the BCA1 kit (Sigma-Aldrich, no. B9643) according to the manufacturer’s instructions. Protein samples were mixed with 5× Laemmli buffer and denatured at 95°C for 5 min. Fifty micrograms of total protein per sample was loaded onto SDS–polyacrylamide gel electrophoresis gels. Proteins were transferred to membranes using the Invitrogen iBlot gel transfer stacks with nitrocellulose membranes (Thermo Fisher Scientific). In some cases, semidry transfer using polyvinylidene difluoride membranes was performed. Antibodies used are listed in Table 1. Primary antibodies were diluted 1:1000 in TBST (Tris-buffered saline with Tween 20) containing either 2% bovine serum albumin (for phosphorylated antibodies) or 2% nonfat dry milk. Secondary antibodies were diluted 1:1000 or 1:5000 in TBST containing 2% nonfat milk.

**Table 1.** List of antibodies.

Antibodies	Source	Catalog no.
Rabbit anti-phospho-mTOR (Ser^2448^)	Cell Signaling Technology	2971S
Rabbit anti-mTOR	Cell Signaling Technology	2972S
Rabbit anti-phospho-p70S6Kinase (T389)	Cell Signaling Technology	9234S
Rabbit anti-p70S6Kinase (49D7)	Cell Signaling Technology	2708S
Rabbit anti-BiP (C50B12)	Cell Signaling Technology	3177S
ECL Donkey Anti-Rabbit IgG, Horseradish Peroxidase	GE Health Care Life Sciences	NA934V
ECL Sheep Anti-Mouse IgG, Horseradish Peroxidase	GE Health Care Life Sciences	NA931V
Rabbit anti-CRY1	Fortis Life Sciences	A302-614A
Rabbit Rev-Erb alpha (E1Y6D)	Cell Signaling Technology	13418
Rabbit anti-BMAL1	Abcam	AB93806
Rabbit anti-phospho-AKT (Ser^473^)	Cell Signaling Technology	4060
Rabbit anti-AKT (pan; C67E7)	Cell Signaling Technology	4691
Mouse anti-alpha tubulin (B-7)	Santa Cruz	sc-5286

### Induction of ER stress by TM

WT mice (C57BL/6) were fed on NF, AF, and AF + AZD8055(night) (*n* = 16 per group) for 2 weeks as described above. Mice were intraperitoneally injected with TM at 1 mg/kg at ZT0, ZT6, ZT12, and ZT18 (*n* = 4 mice/group/time point) and euthanized 4 hours after injection to collect the liver. Livers were flash-frozen in liquid nitrogen for storage and later use.

### Analysis of hepatic effect of ER stress

To determine the effect of ER stress induction in the three groups WT-NF-ctrl, WT-AF, and WT-AF-AZDnight, raw gene expression from the TM-treated and corresponding control samples was normalized together using the dryR package (i.e., WT-NF-ctrl was normalized with WT-NF-ctrl-TM, WT-AF with WT-AF-TM, and WT-AF-AZDnight with WT-AF-AZDnight-TM). DEGs in each group were then identified using the DESeq2 package (*104*). Genes with an adjusted *P* value (*P*_adj_) ≤0.05 and a fold change >1.5 were considered differentially expressed.

To assess potential time-of-day differences in the response to ER stress within each group, the top 1000 DEGs across all groups and time points were identified using the DESeq2 package by comparing all TM-treated samples (*n* = 48) to all control samples (*n* = 54 untreated WT-NF-ctrl mice, WT-AF mice, and WT-AF-AZDnight mice). The 1000 DEGs with the highest fold change were selected, and the fold change was computed for these 1000 genes for each group at ZT10 and ZT22 (the two time points common between control and TM-treated samples). Statistical differences in fold change between time points across the three groups were assessed using a two-way analysis of variance (ANOVA). *P* ≤ 0.05 was considered statistically significant. To detect and compare rhythmicity in TM-treated groups, all three groups were normalized together using the dryR package, and rhythmicity analysis was performed as previously described.

Last, to assess how the manipulation of rhythmic mTOR activity affects UPR target genes, we used public datasets that identified ER stress–induced target genes for the TFs ATF4 (*60*), ATF6 (*61*), and drug-induced XBP1s (*62*). For ATF4 target genes, we directly used the list of genes significantly increased with TM in WT mouse embryonic fibroblasts (*P* < 0.05) but not ATF4 knockout mouse embryonic fibroblasts that was provided in the study (*60*). For ATF6 target genes, we applied a fold change threshold (FC ≥ 1.5) to the list of genes affected by ER stress in human embryonic kidney 293 cells (*61*). Last, for drug-induced XBP1s target genes, we only used genes significantly increased (FC ≥ 1.5 and *P*_adj_ ≤ 0.05) in the livers of diet-induced obesity mice treated with IXA4 (XBP1s inducer) compared to vehicle-treated mice (*62*).

### Global metabolomics profiling

The metabolomic analysis was entirely performed by Metabolon (Durham, NC). Liver samples were collected at six time points (ZT2, ZT6, ZT10, ZT14, ZT18, and ZT22) in WT mice exposed to different feeding paradigms or treatment conditions. The three groups were NF (WT-NF), AF (WT-AF), and AF with AZD8055 injection (WT-AF-AZDnight) administered at ZT11.5. Each group includes three replicates per time point (*n* = 18 per group), and each replicate is a mixture of two liver sample replicates. Original data provided by Metabolon are provided in table S10 and processed data in table S11.

### Sample preparation

Samples were prepared using the automated MicroLab STAR system from Hamilton Company. Several recovery standards were added before the first step in the extraction process for quality control (QC) purposes. To remove protein, small molecules bound to protein or trapped in the precipitated protein matrix were dissociated, and to recover chemically diverse metabolites, proteins were precipitated with methanol under vigorous shaking for 2 min (Glen Mills GenoGrinder 2000) followed by centrifugation. The resulting extract was divided into five fractions: two for analysis by two separate reverse phase (RP)/ultrahigh-performance liquid chromatography–tandem mass spectroscopy (UPLC-MS/MS) methods with positive ion mode electrospray ionization (ESI), one for analysis by RP/UPLC-MS/MS with negative ion mode ESI, one for analysis by HILIC (hydrophilic interaction chromatography)/UPLC-MS/MS with negative ion mode ESI, and one sample reserved for backup. Samples were placed briefly on a TurboVap (Zymark) to remove the organic solvent. The sample extracts were stored overnight under nitrogen before preparation for analysis.

### Quality assurance/quality control

Several types of controls were analyzed in concert with the experimental samples: A pooled matrix sample generated by taking a small volume of each experimental sample (or, alternatively, use of a pool of well-characterized human plasma) served as a technical replicate throughout the dataset, extracted water samples served as process blanks, and a cocktail of QC standards that were carefully chosen not to interfere with the measurement of endogenous compounds was spiked into every analyzed sample, allowed instrument performance monitoring, and aided chromatographic alignment. Instrument variability was determined by calculating the median relative standard deviation for the standards that were added to each sample before injection into the mass spectrometers. Overall process variability was determined by calculating the median relative standard deviation for all endogenous metabolites (i.e., noninstrument standards) present in 100% of the pooled matrix samples. Experimental samples were randomized across the platform run with QC samples spaced evenly among the injections.

### Ultrahigh-performance liquid chromatography–tandem mass spectroscopy (UPLC-MS/MS)

All methods used a Waters ACQUITY ultraperformance liquid chromatography (UPLC) system and a Thermo Fisher Scientific Q-Exactive high-resolution/accurate mass spectrometer interfaced with a heated ESI (HESI-II) source and Orbitrap mass analyzer operated at 35,000 mass resolution. The sample extract was dried and then reconstituted in solvents compatible to each of the four methods. Each reconstitution solvent contained a series of standards at fixed concentrations to ensure injection and chromatographic consistency. One aliquot was analyzed using acidic positive ion conditions, chromatographically optimized for more hydrophilic compounds. In this method, the extract was gradient eluted from a C18 column (Waters UPLC BEH C18-2.1 × 100 mm, 1.7 μm) using water and methanol containing 0.05% perfluoropentanoic acid and 0.1% formic acid. Another aliquot was also analyzed using acidic positive ion conditions; however, it was chromatographically optimized for more hydrophobic compounds. In this method, the extract was gradient eluted from the same aforementioned C18 column using methanol, acetonitrile, water, 0.05% perfluoropentanoic acid, and 0.01% formic acid and was operated at an overall higher organic content. Another aliquot was analyzed using basic negative ion–optimized conditions using a separate dedicated C18 column. The basic extracts were gradient eluted from the column using methanol and water, however, with 6.5 mM ammonium bicarbonate at pH 8. The fourth aliquot was analyzed via negative ionization following elution from a HILIC column (Waters UPLC BEH Amide 2.1 × 150 mm, 1.7 μm) using a gradient consisting of water and acetonitrile with 10 mM ammonium formate, pH 10.8. The MS analysis alternated between MS and data-dependent MS^n^ scans using dynamic exclusion. The scan range varied slightly between methods but covered 70 to 1000 *m*/*z* (mass/charge ratio).

### Data extraction, compound identification, and metabolite quantification and data normalization

Raw data were extracted, peak identified, and QC processed using Metabolon’s hardware and software. These systems are built on a web-service platform using Microsoft’s .NET technologies, which run on high-performance application servers and fiber-channel storage arrays in clusters to provide active failover and load balancing. Compounds were identified by comparison to library entries of purified standards or recurrent unknown entities. Biochemical identifications are based on three criteria: retention index within a narrow retention index window of the proposed identification, accurate mass match to the library +/− 10 ppm (parts per million), and the MS/MS forward and reverse scores between the experimental data and authentic standards. The MS/MS scores are based on a comparison of the ions present in the experimental spectrum to the ions present in the library spectrum. Peaks were quantified using the area under the curve. For studies spanning multiple days, a data normalization step was performed to correct variation resulting from instrument interday tuning differences.

### Rhythmicity analysis of metabolome

Rhythmicity analysis was performed on imputed data from Metabolon (with a cutoff including samples with ≤6 missing values) as described above for transcript rhythmicity, with minor modifications. First, six programs were used to test for rhythmicity: JTK-CYCLE, ARSER, LS (*105*), F24, RAIN, and Harmonic Regression. Second, the resulting *P* values from all six programs were combined using the Harmonic mean of *P* value method, which was then used to calculate the number of rhythmic metabolites. Metabolites with *P* ≤ 0.05 were considered significantly rhythmic.

## Supplementary Materials

### The PDF file includes:

Figs. S1 to S20 Uncropped Western blots Legends for tables S1 to S11
