# Supplementary material for: mTOR signaling contributes to system-driven rhythmic gene expression in mouse liver
Source: Sci Adv. 2026 Jul 23;12(30):eaec0131. doi: 10.1126/sciadv.aec0131 (PMC13394481; doi:10.1126/sciadv.aec0131)
Supplement: Supplementary file 1 — Figs. S1 to S20 Uncropped Western blots Legends for tables S1 to S11 [file sciadv.aec0131_sm.pdf]

Science Advances
AAAS

Supplementary Materials for

mTOR signaling contributes to system-driven rhythmic gene expression in mouse liver

Aishwarya Sahasrabudhe *et al.*

Corresponding author: Jerome S. Menet, menet@tamu.edu

Sci. Adv. **12**, eaec0131 (2026)
DOI: 10.1126/sciadv.aec0131

The PDF file includes:

Figs. S1 to S20
Uncropped Western blots
Legends for tables S1 to S11

Other Supplementary Material for this manuscript includes the following:

Tables S1 to S11

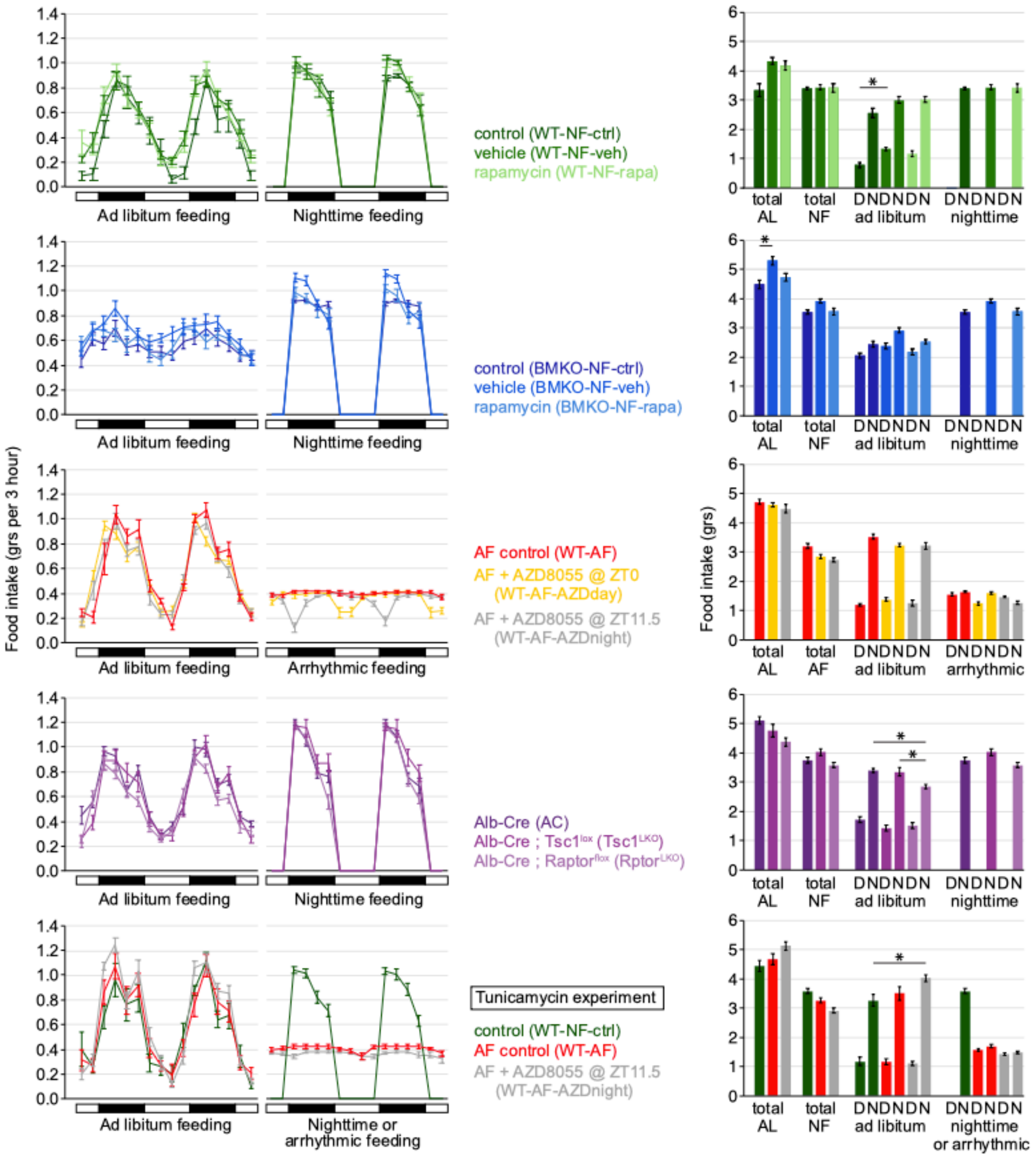

Fig. S1: Effect of the feeding paradigms and drug treatments on food/calorie intake in mice (Left): Food intake profiles across two days of *ad libitum* feeding and two days of nighttime or arrhythmic feeding in all groups of mice, binned per 3 hours. Values represent the mean ± SEM; n = 16-18 per group. (Right): Total food intake (grams) under the different feeding paradigms, represented as daytime versus nighttime food intake (grams) across the feeding paradigms or drug treatments in the different groups of mice. Values represent the mean ± SEM; n = 16-18 per group. Asterisks illustrate significant differences between groups ($p < 0.05$; 3-way ANOVA). Differences between daytime and nighttime feeding, which are significantly different in all nighttime-fed or *ad libitum*-fed mice but not in arrhythmically-fed mice, are not shown.

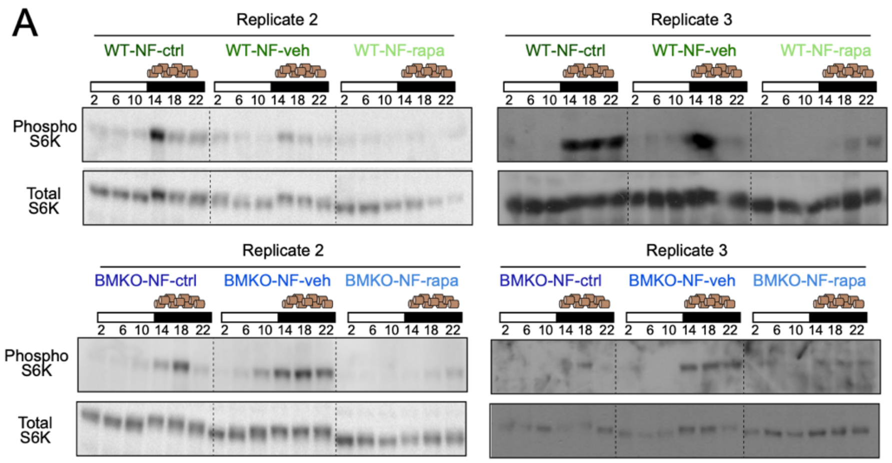

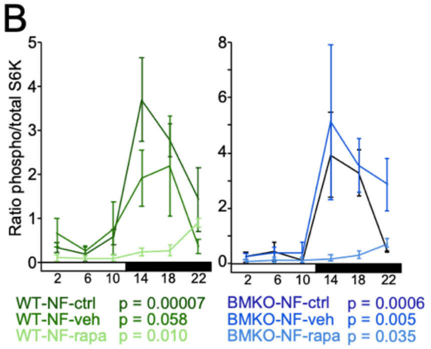

C

	JTK	F24	RAIN	HaRE	hmp	BH
WT-NF-ctrl	3573	4978	5902	4906	5293	3377
WT-AF-ctrl	2301	3203	4575	3149	3712	1560
BMKO-NF-ctrl	2024	2845	4013	2469	3323	1222
WT-NF-veh	2965	4240	5344	4178	4660	2622
WT-NF-rapa	1957	2764	3636	2588	3066	1149
BMKO-NF-veh	1926	2909	3752	2827	3195	1316
BMKO-NF-rapa	1002	1688	2650	1620	1994	130
AC-NF	4075	5235	6467	5089	5729	3979
$Tsc1^{LKO}$-NF	3835	5172	6177	5034	5509	3814
$Rptor^{LKO}$-NF	2980	4053	5074	3980	4479	2626
WT-AF-AZDday	2885	4164	5757	4020	4859	2849
WT-AF-AZDnight	2992	4357	5670	4204	4921	2861

Fig. S2: mTOR rhythmicity regulates rhythmic gene expression in the mouse liver
(A) Western blots of phospho-S6K and total S6K levels in mouse liver (additional replicates to that shown in Fig. 1B). **(B)** Western blot quantification, with n = 3 independent biological replicates per time point. p-values were calculated as the harmonic mean p-values from 4 rhythmicity tests. **(C)** Table displaying the number of rhythmic genes calculated by four different rhythmicity tests (JTK-cycle, F24, RAIN and Harmonic Regression -HaRE-), the harmonic mean of p-values (hmp), and the Benjamini-Hochberg (BH) adjusted p-values (q-values), computed on 12 different groups.

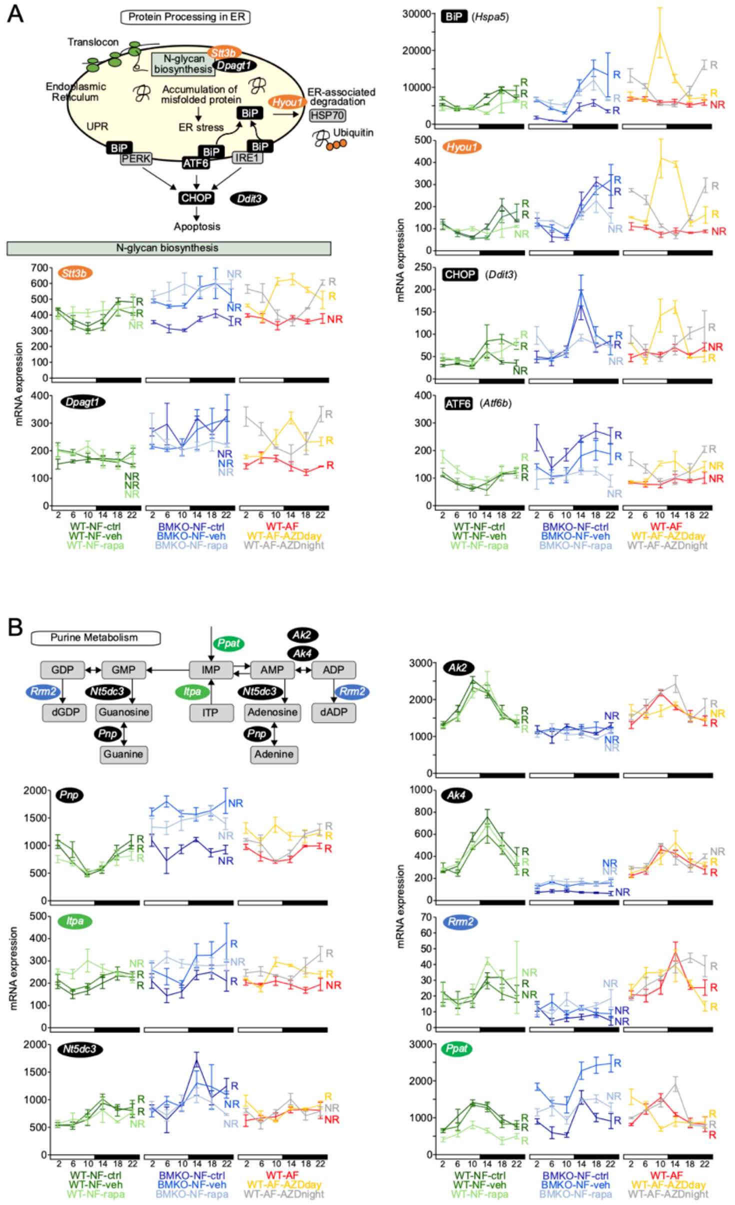

Fig. S3: Expression profiles of representative genes within the KEGG pathways 'protein processing in the ER' and 'purin metabolism'

Expression of genes associated with protein processing in endoplasmic reticulum (ER) and N-glycan biosynthesis pathways **(A)** and purine metabolism pathway **(B)**, illustrating the impact of arrhythmic feeding and mTOR inhibition on their expression profiles. Values represent the mean ± SEM of n = 3 independent biological replicates per time point. Rhythmic expression was calculated using the harmonic mean p-values from 4 rhythmicity tests. R: rhythmic expression ($p \leq 0.05$). NR: non rhythmic expression ($p > 0.05$). Clock-driven rhythmic genes are colored in blue, mTOR-driven rhythmic genes in orange, clock- or mTOR-driven rhythmic genes in green, and clock- and mTOR-driven rhythmic genes in purple, as per Fig. 4D categorization. Genes colored in black were not assigned to any of these 4 groups yet exhibit some regulation by the clock and/or mTOR.

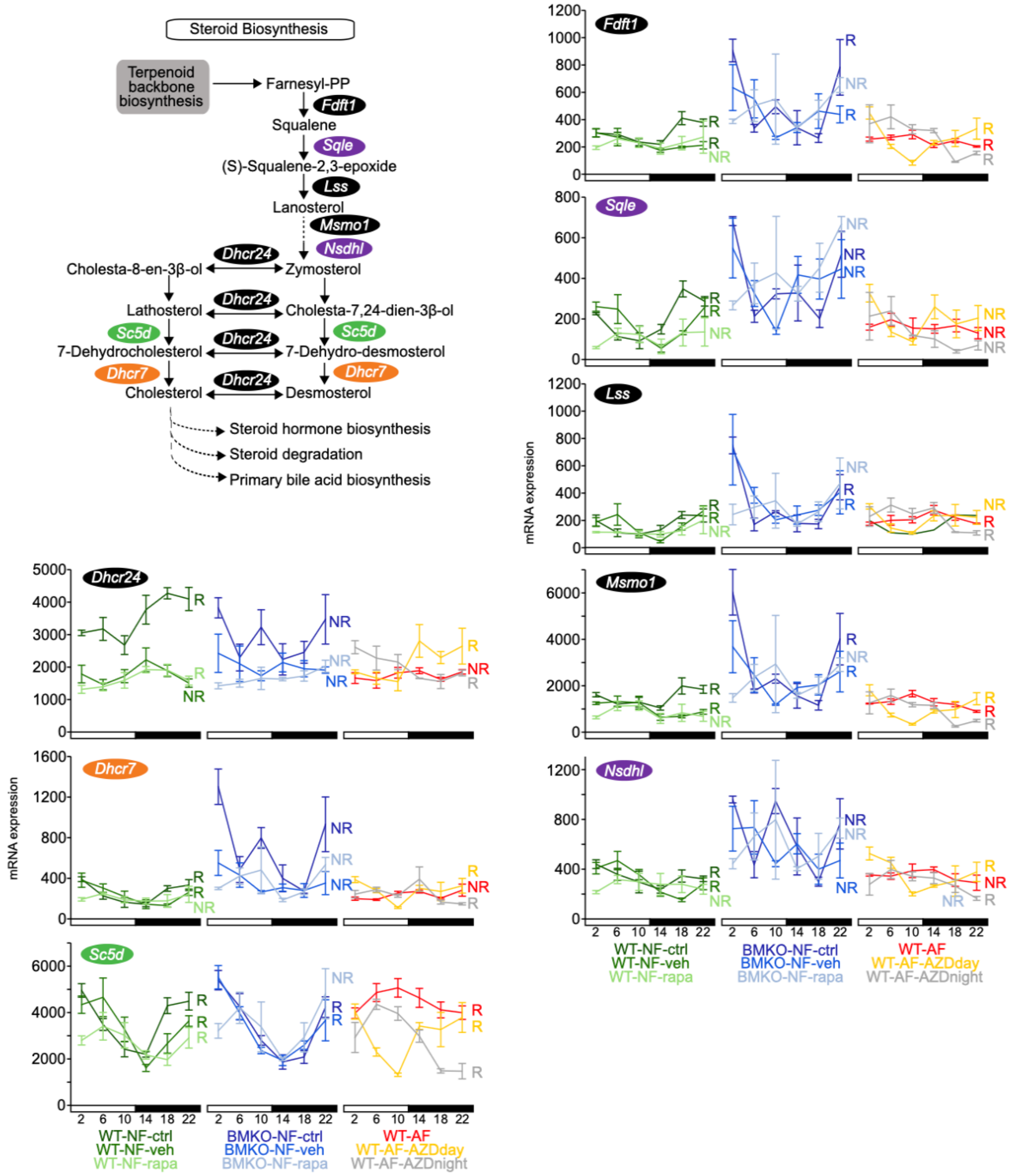

Fig. S4: Expression profiles of representative genes within the KEGG pathways ‘steroid biosynthesis’

Expression of genes associated with the steroid biosynthesis pathway, illustrating the impact of arrhythmic feeding and mTOR inhibition on their expression profiles. Values represent the mean ± SEM of n = 3 independent biological replicates per time point. Rhythmic expression was calculated using the harmonic mean p-values from 4 rhythmicity tests. R: rhythmic expression ($p \leq 0.05$). NR: non rhythmic expression ($p > 0.05$). Clock-driven rhythmic genes are colored in blue, mTOR-driven rhythmic genes in orange, clock- or mTOR-driven rhythmic genes in green, and clock- and mTOR-driven rhythmic genes in purple, as per Fig. 4D categorization. Genes colored in black were not assigned to any of these 4 groups yet exhibit some regulation by the clock and/or mTOR.

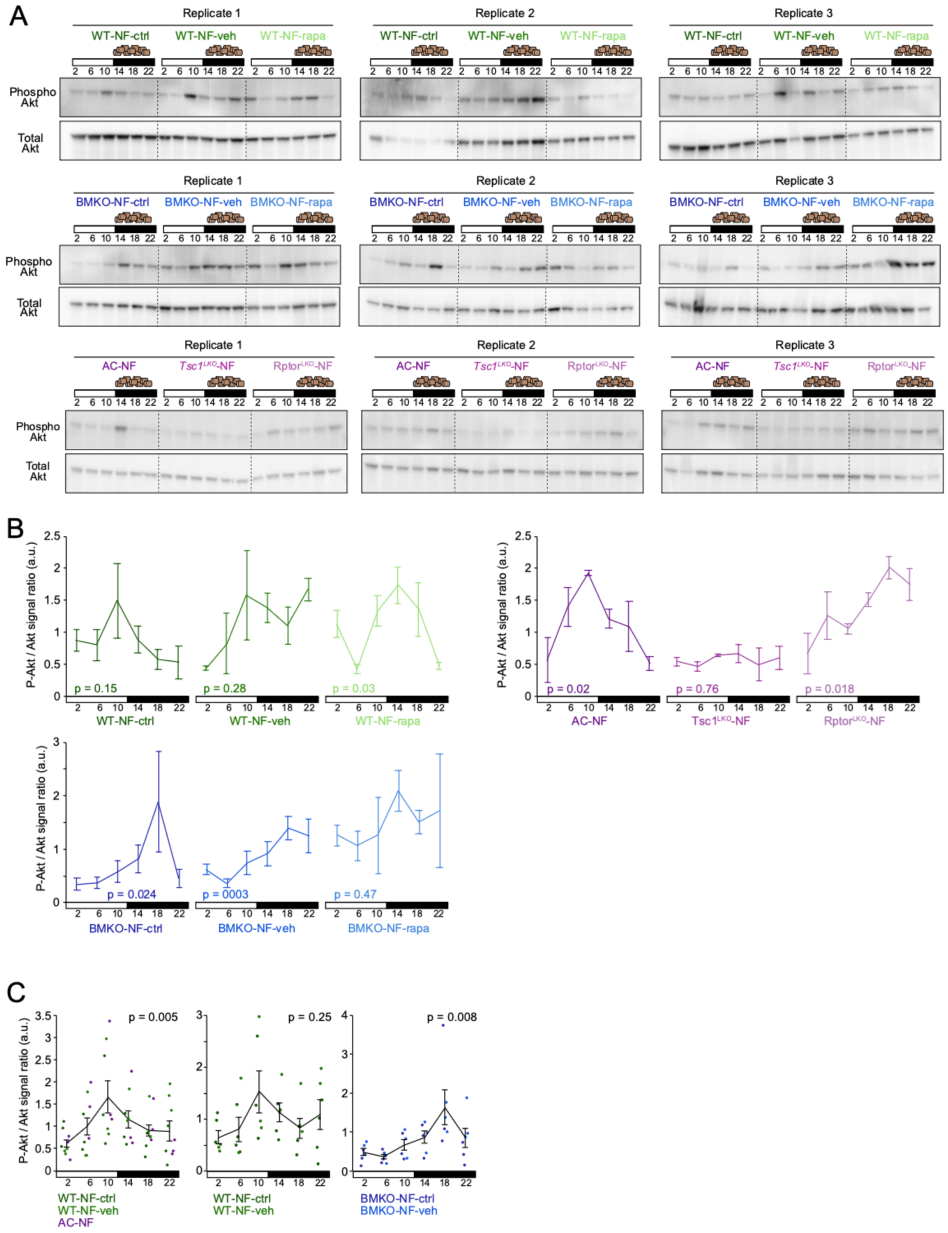

Fig. S5: Effect of mTOR inhibition on mTORC2 activity.

(A) Western blots of AKT phosphorylation at S473, a canonical mTORC2 target site, and total AKT in the liver of WT-NF-ctrl, WT-NF-veh, WT-NF-rapa, BMKO-NF-ctrl, BMKO-NF-veh, BMKO-NF-rapa, *Alb-Cre*-NF, Tsc1^{LKO}-NF and Rptor^{LKO}-NF mice. The three replicates depict independent biological replicates for each group. **(B, C)** Western blot quantification of hepatic AKT activity, calculated as the ratio between phospho-S473 AKT and total AKT. p-values were calculated as the harmonic mean p-values from 4 rhythmicity tests.

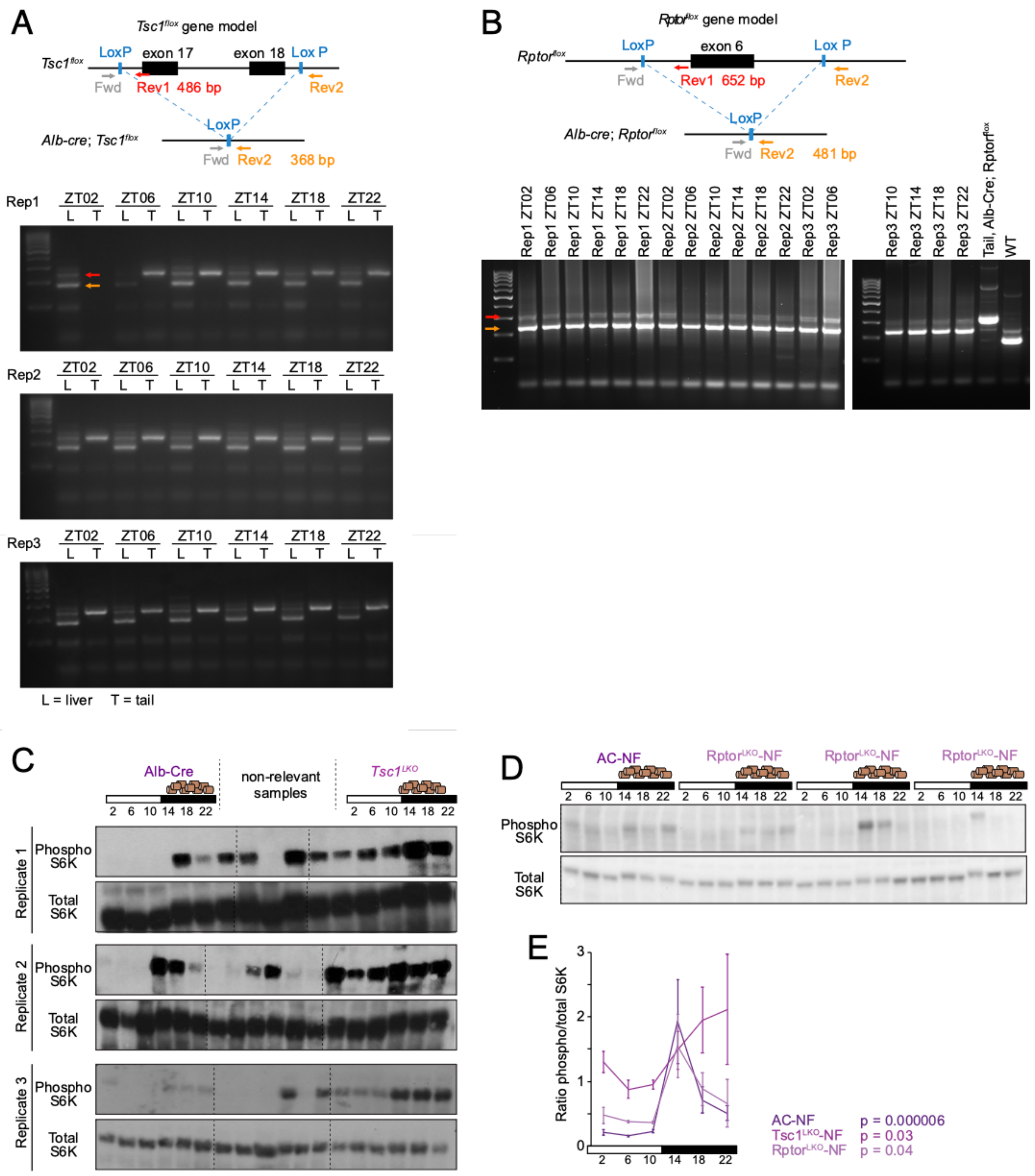

Fig. S6: Characterization of Tsc1^{LKO} and Rptor^{LKO} mice.
(A) Schematic of *Tsc1^{flox}* gene model (top) and agarose gel images of genotyping from liver and tail snips confirming liver specific excision (bottom). **(B)** Schematic of *Rptor^{flox}* gene model (top) and agarose gel images of genotyping from liver and tail snips confirming liver specific excision (bottom). **(C,D)** Western blot analysis of phospho-S6K and total S6K levels in mouse liver of Alb-Cre-NF and Tsc1^{LKO}-NF mice (C) and of Alb-Cre-NF and Rptor^{LKO}-NFmice (D). The blots for replicate 3 of Alb-Cre-NF and Tsc1^{LKO}-NF mice are also partly shown in Fig. 2E, and show that

the signals for the two genotypes originate from the same blot and with the same exposure time. **(E)** Western blot quantification of S6K phosphorylation, calculated as the ratio between phospho-S6K and total S6K. p-values were calculated as the harmonic mean p-values from 4 rhythmicity tests.

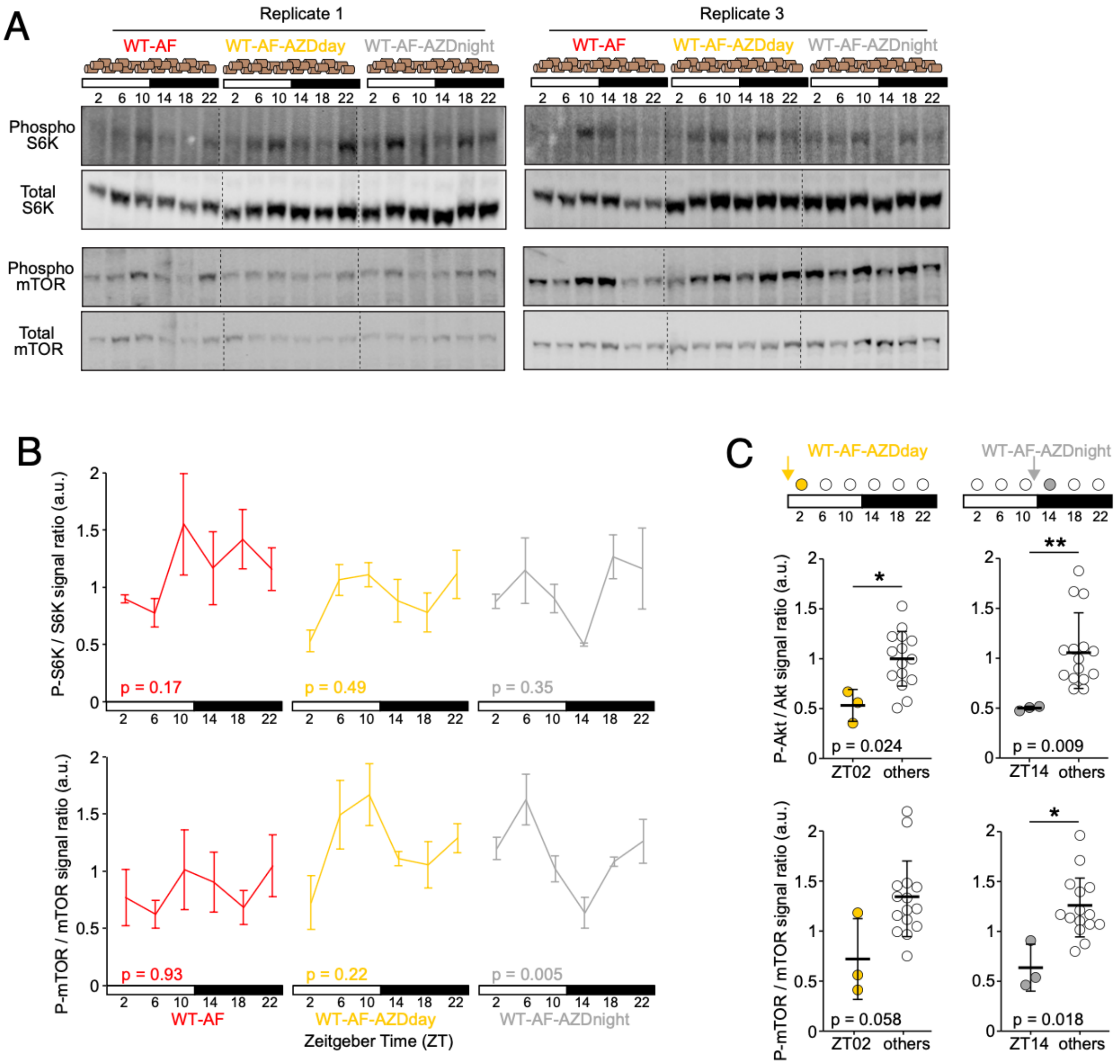

Fig. S7: Effect of the short half-life mTOR inhibitor AZD8055 on S6K and mTOR phosphorylation.

(A) Western blots of phospho-S6K, total S6K, phospho-mTOR, and total mTOR in mouse liver after AZD8055 treatment in arrhythmically fed mice (additional replicates to those shown in Fig. 3C). **(B)** Western blot quantification, with n = 3 independent biological replicates per time point. Error bars correspond to the SEM of the 3 replicates. p-values were calculated as the harmonic mean p-values from 4 rhythmicity tests. **(C)** Quantification of phospho-S6K and phospho-mTOR levels 2 hours post-AZD8055 injection (closed circles) and later time points (open circles). Error bars represent the mean +/- standard deviation. P-values were calculated by the Mann-Whitney U test, and considered significant if $p \leq 0.05$.

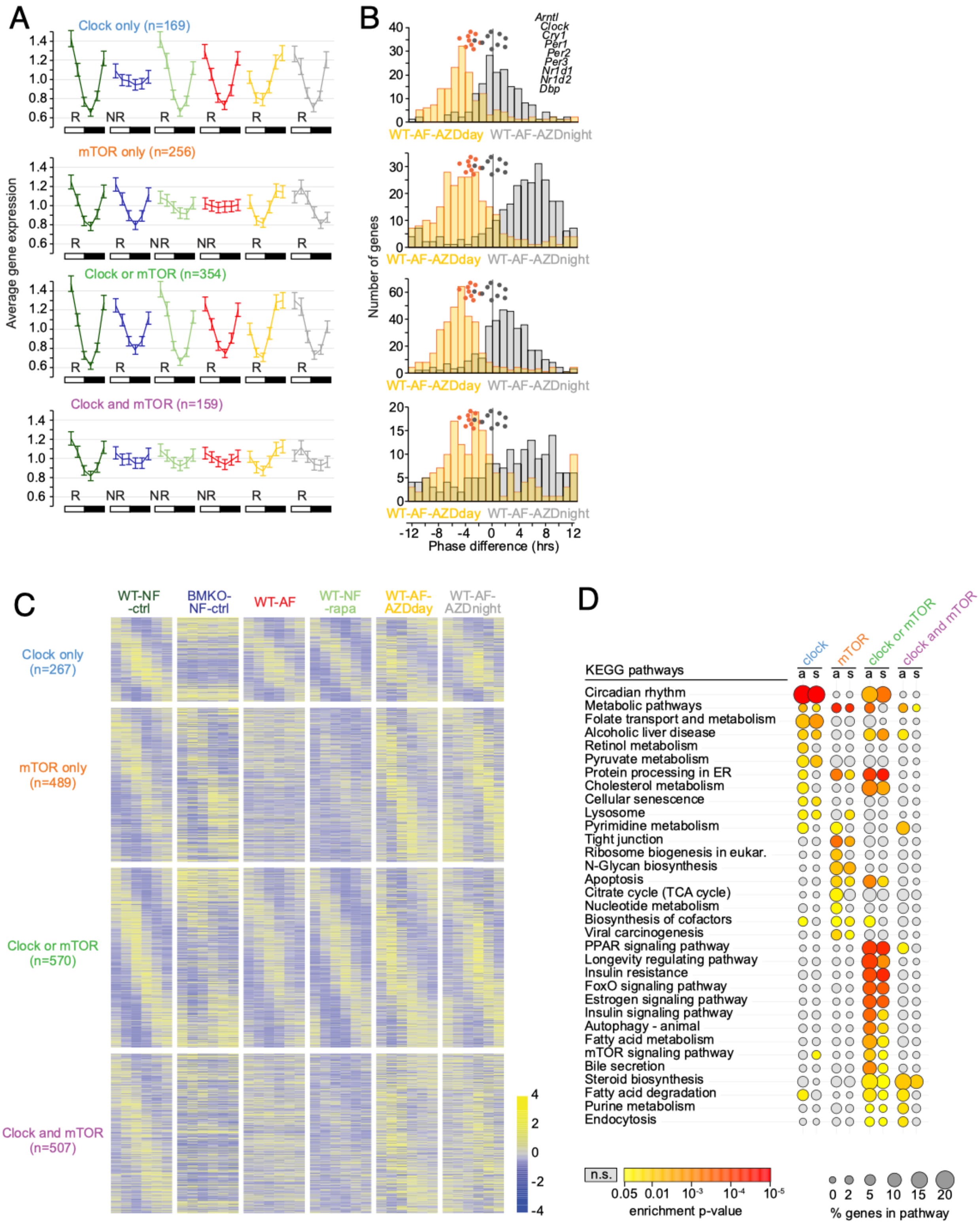

Fig. S8: Distinct categories of transcripts show different rhythmicity profiles across groups.

(A) Average gene expression profile according to stringent analysis of clock-driven, mTOR-driven, clock- or mTOR-driven, and clock- and mTOR-driven rhythmic genes in different groups of mice (WT-NF-ctrl: dark green; BMKO-NF-ctrl: blue; WT-NF-rapa: light green; WT-AF: red; WT-AF-AZDday: yellow; WT-AF-AZDnight: grey). Values represent mean ± SEM; n=3 per time point. **(B)** Phase difference between WT-NF-ctrl and WT-AF-AZDday mice (yellow) and between WT-NF-ctrl and WT-AF-AZDnight mice (grey), for clock-driven, mTOR-driven, clock- or mTOR-driven, and clock- and mTOR-driven rhythmic genes using stringent cut-off. Negative values represent a phase-advance in AZD8055-treated mice while positive values represent a phase delay. The yellow and grey dots represent core clock genes as labelled. **(C)** Heatmap illustrating the rhythmic expression of clock-driven, mTOR-driven, clock- or mTOR-driven, and clock- and mTOR-driven rhythmic genes (based on less stringent analysis) across 6 groups (WT-NF-ctrl, BMKO-NF-ctrl, WT-AF, WT-NF-rapa, WT-AF-AZDday, WT-AF-AZDnight). Data in each column is the averaged signal by time point (n=3). **(D)** KEGG pathway enrichment analysis of genes across 4 different groups (clock-driven, mTOR-driven, clock- or mTOR-driven, and clock- and mTOR-driven rhythmic genes) carried out using the complete gene list (a) and the stringent gene list (s). Selected enriched KEGG pathways are represented with the size of the circle representing the percentage of rhythmic genes in the pathway and the color scale indicating p-value enrichment (-log10).

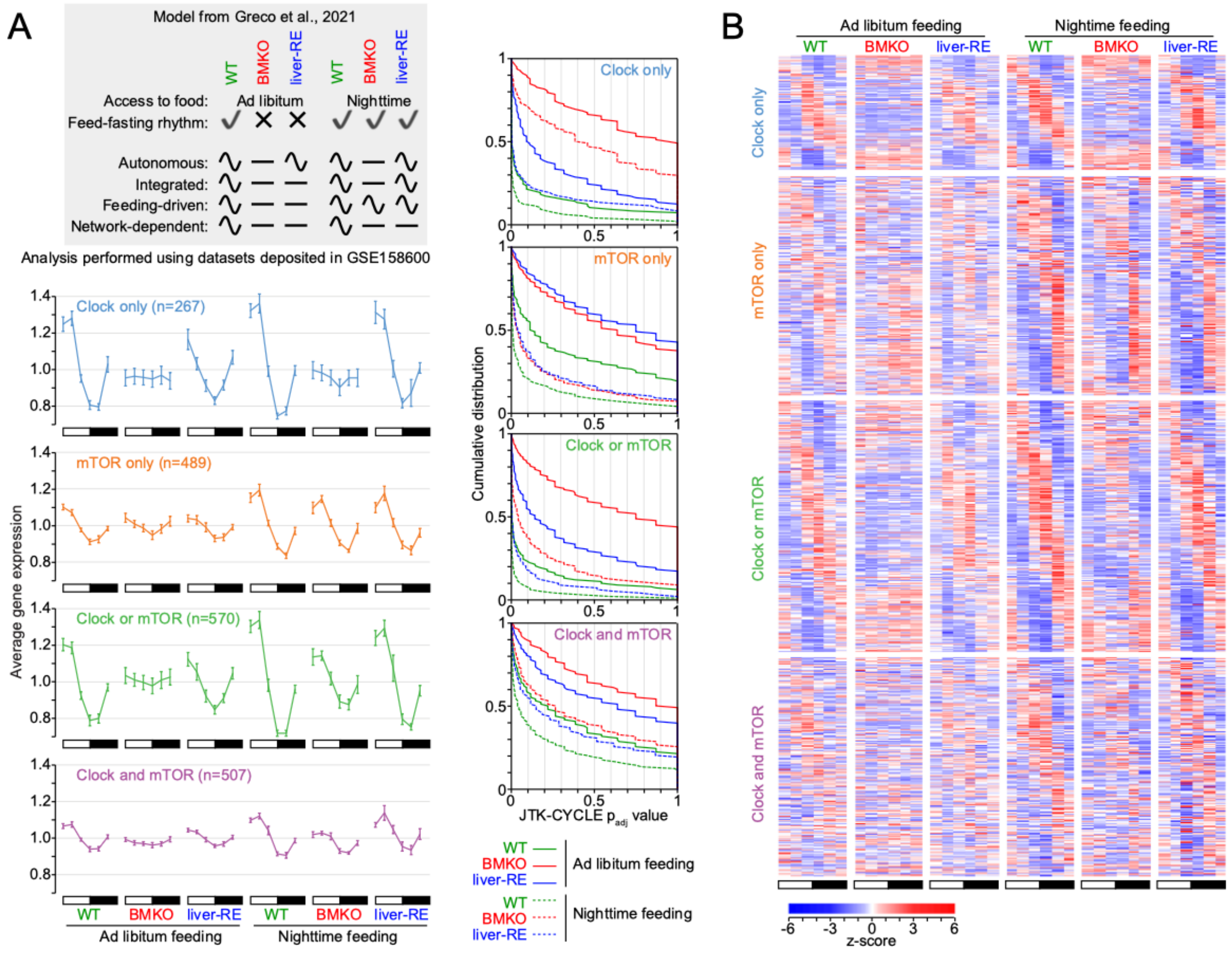

Fig. S9: Expression profiles of mTOR-driven and clock-driven genes relative to central/systemic cues, autonomous liver clock and feeding-driven signals
(A) (Left): Average gene expression profile of clock-driven, mTOR-driven, clock- or mTOR-driven, and clock- and mTOR-driven rhythmic genes across different genotypes of mice established in references *(42,43)* (WT: green; BMKO: red; liver-specific *Bmal1* rescue (liver-RE): blue) under either *ad libitum* or nighttime feeding. Values represent mean ± SEM; n=3 per time point. Publicly available liver RNA-seq dataset GSE158600 was used for analysis. (Right)**:** Cumulative distribution of p_{adj}-values (from JTK-cycle) for the same six groups of mice. **(B)** Heatmap illustrating the expression of clock-driven, mTOR-driven, clock- or mTOR-driven, and clock- and mTOR-driven rhythmic genes across 6 groups (WT, BMKO, and liver-RE under *ad libitum* or nighttime feeding. Data in each column corresponds to the averaged signal by time point (n = 3).

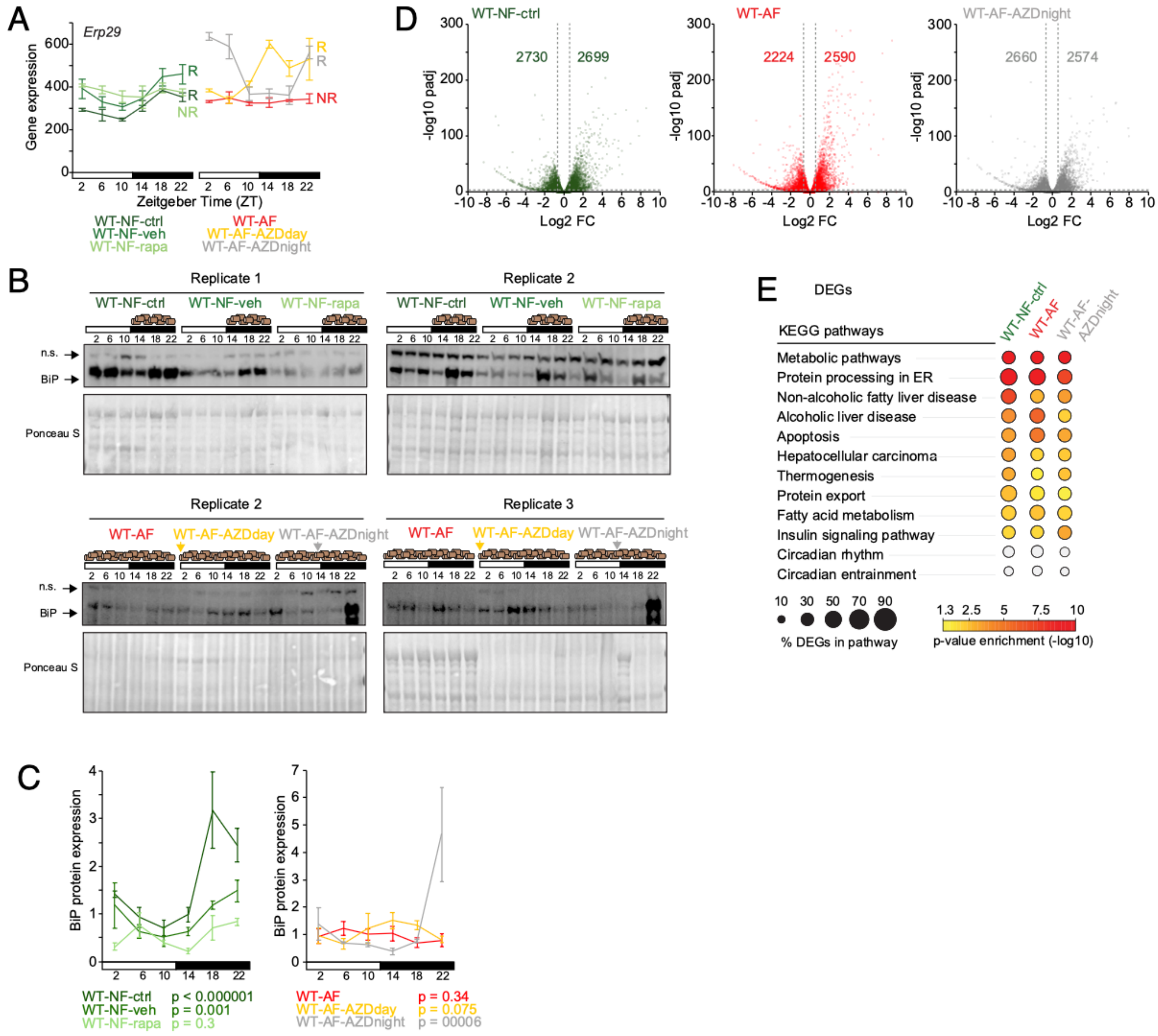

Fig. S10: Contribution of mTOR rhythmic activity to the protein processing in the ER pathway and ER stress response

(A) Gene expression profile of the ER chaperone *Erp29* in the liver of W52T-NF-ctrl, WT-NF-veh, WT-NF-rapa, WT-AF, WT-AF-AZDday and WT-AF-AZDnight mice. Values represent mean ± SEM; n=3 per time point. **(B)** Western blot analysis of BiP expression in mouse liver in different groups. Ponceau stains of the same membrane are displayed below BiP western blots. The asterisk denotes a non-specific (n.s.) band on the BiP western blot. **(C)** Quantification of BiP levels in liver based on Western blot signals, calculated as the ratio between BiP levels and Ponceau stain intensity, with n = 3 independent biological replicates per time point. Error bars correspond to the SEM of the 3 replicates. p-values were calculated as the harmonic mean p-values from 4 rhythmicity tests **(D)** Volcano plot representing fold-change in hepatic gene expression after TM injection in WT-NF-ctrl, WT-AF, and WT-AF-AZDnight mice. The number of DEGs between TM injected and control mice was calculated using the average expression across all time points (n = 18 for control mice, n = 16 for TM injected mice), considering a fold-

change ≥ 1.5, and p_{adj} ≤ 0.05. **(E)** KEGG pathway enrichment analysis of genes of DEGs after TM injection.

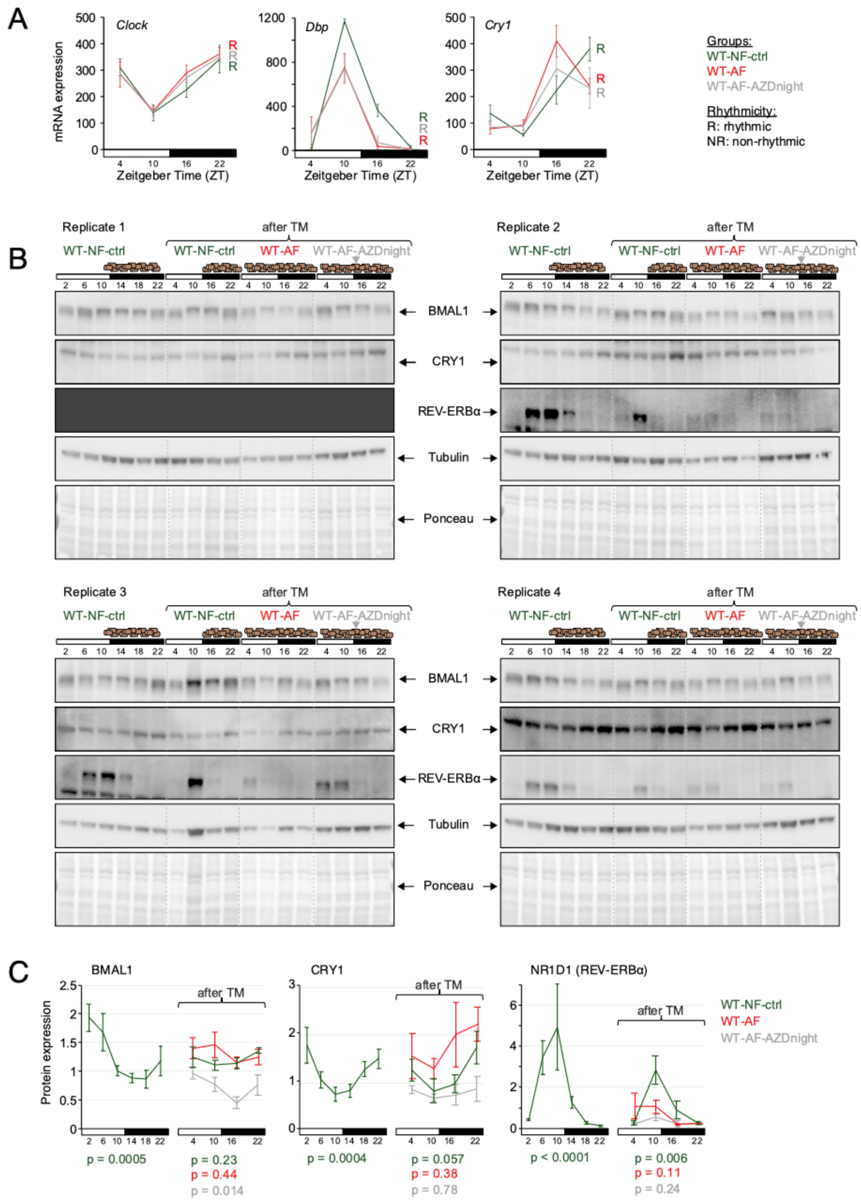

Fig. S11: Effect of TM-induced ER stress of the hepatic expression of a core clock genes (A) Gene expression profiles of three clock genes (*Clock*, *Dbp*, *Cry1*) in the liver of WT-NF-ctrl non-treated with tunicamyin (TM) (6 time points), as well as in WT-NF-ctrl, WT-AF, and WT-AF-AZDnight mice after TM injection (4 time points). Values represent the mean ± SEM of n = 3-4 independent biological replicates per time point. **(B)** Western blot analysis of the core clock proteins BMAL1, CRY1 and REV-ERBα in mouse liver in different groups after TM injection. **(C)** Quantification of core clock proteins levels in mouse liver after ER stress induction, based on Western blot signals. Protein expression was calculated as the ratio between clock protein levels and Ponceau stain intensity, and is displayed as the mean ± SEM of n = 3-4 independent biological replicates per time point. p-values were calculated as the harmonic mean p-values from 4 rhythmicity tests.

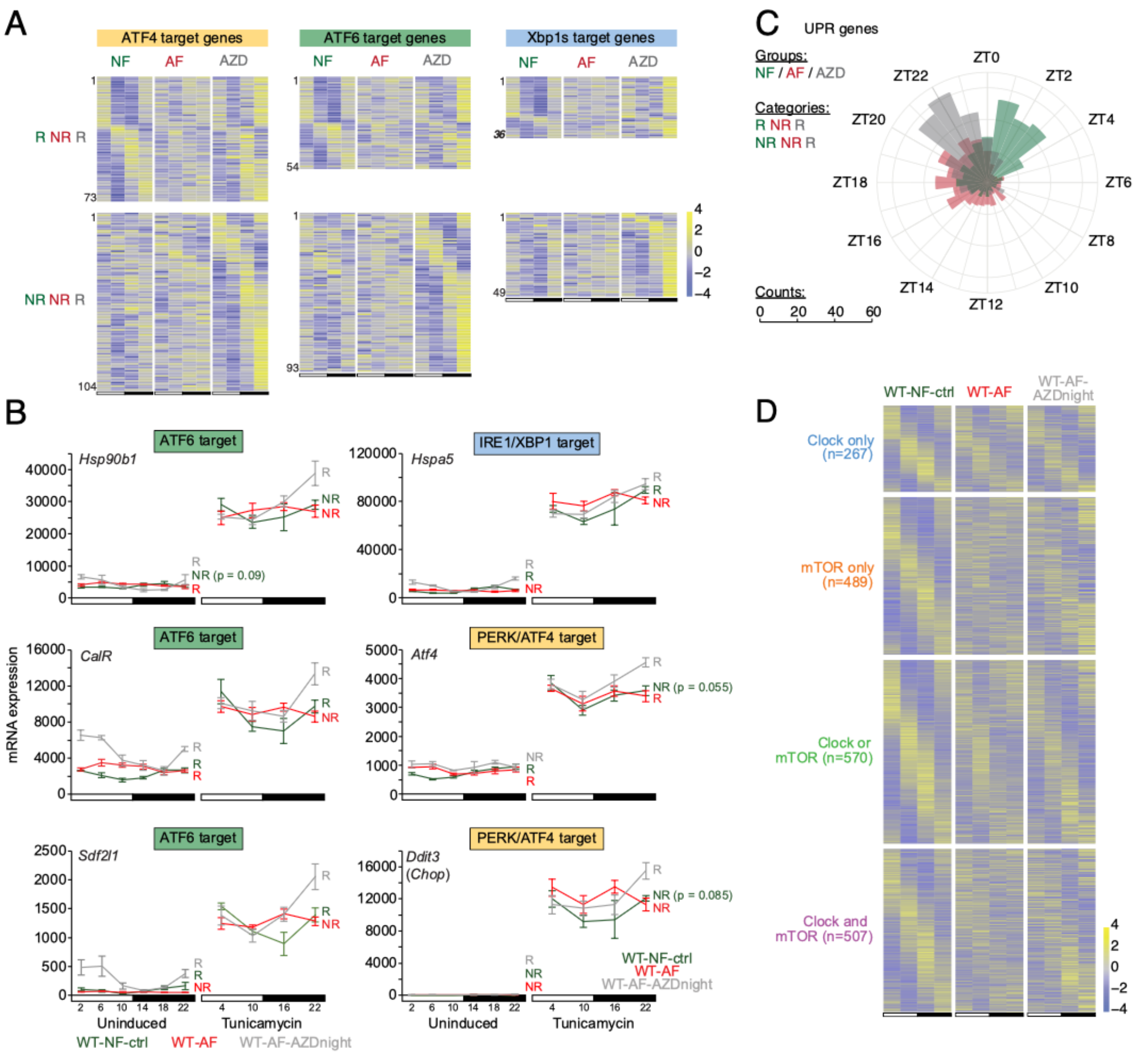

Fig. S12: mTOR signaling regulates the unfolded protein response (UPR) pathway
(A) Heatmap illustrating how manipulation of mTOR rhythmic activity modulates the expression of genes associated with the unfolded protein response (UPR) pathway, and being targeted by the transcription factors ATF4, ATF6, and XBP1s. **(B)** Gene expression profiles of ER stress response genes in uninduced (left) and TM-injected (right) mice. Values represent the mean ± SEM of n=3 biological replicates per time point for uninduced mice and n = 4 biological replicates for TM-injected mice. R: rhythmic gene. NR: non-rhythmic gene. The p-value of NR genes is displayed if $p < 0.1$. **(C)** Rose plot of UPR genes. **(D)** Heatmap illustrating the rhythmic expression of clock-driven, mTOR-driven, clock- or mTOR-driven, and clock- and mTOR-driven rhythmic genes (based on less stringent analysis) after TM injection in WT-NF-ctrl, WT-AF, and WT-AZDnight. Data in each column correspond to the averaged signal by time point (n = 4).

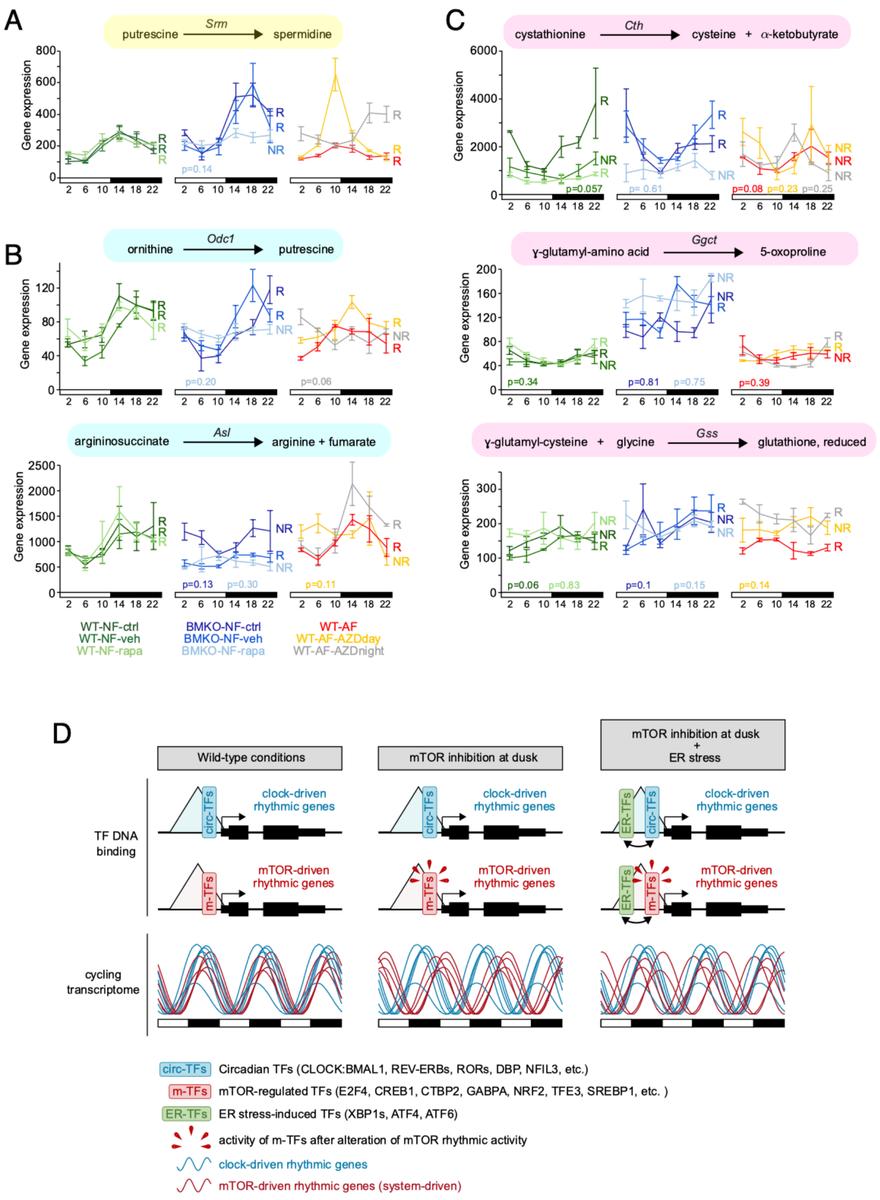

Fig. S13: Expression of some enzymes and their partial regulation by mTOR rhythmic activity in mouse liver

(A-C) Expression profile of spermidine synthase (*Srm*), ornithine decarboxylase 1 (*Odc1*), argininosuccinate lyase (*Asl*), cystathionine gamma-lyase (*Cth*), γ-glutamylcyclotransferase (*Ggct*), and glutathione synthetase (*Gss*) in the liver of WT-NF-ctrl, WT-NF-veh, WT-NF-rapa, BMKO-NF-ctrl, BMKO-NF-veh, BMKO-NF-rapa, WT-AF, WT-AF-AZDday and WT-AF-AZDnight mice. Values represent mean ± SEM; n=3 per time point. **(D)** Model of how clock-driven TFs, mTOR-driven TFs and ER stress activated TFs may interact to generate the transcriptional output observed after tunicamycin treatment, as shown in Fig. 6.

Western Blot from Fig. 1B and S2A

WT-NF-ctrl / WT-NF-veh / WT-NF-rapa

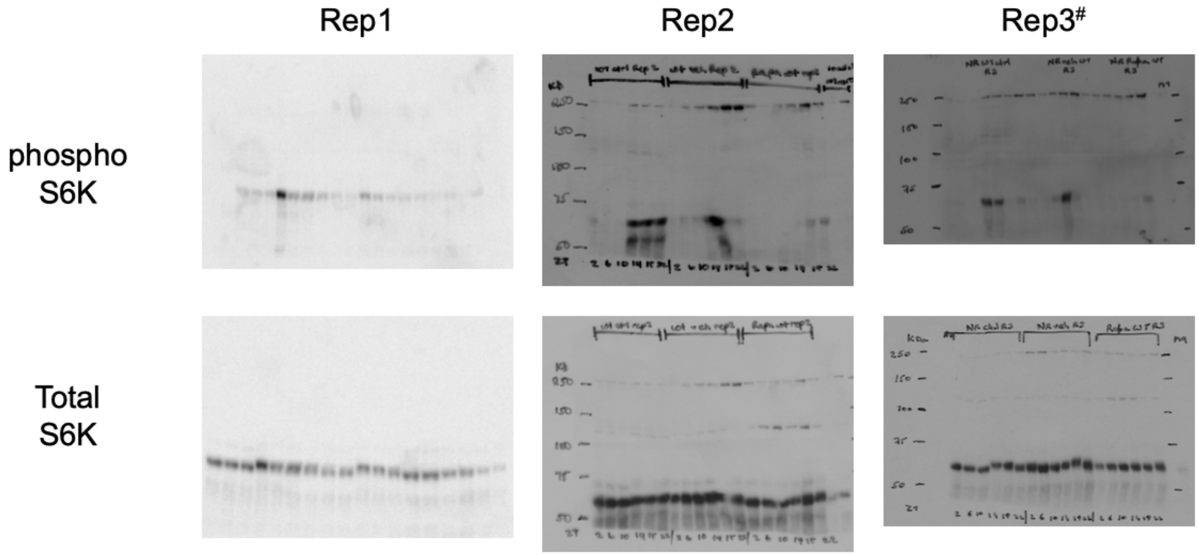

BMKO-NF-ctrl / BMKO-NF-veh / BMKO-NF-rapa

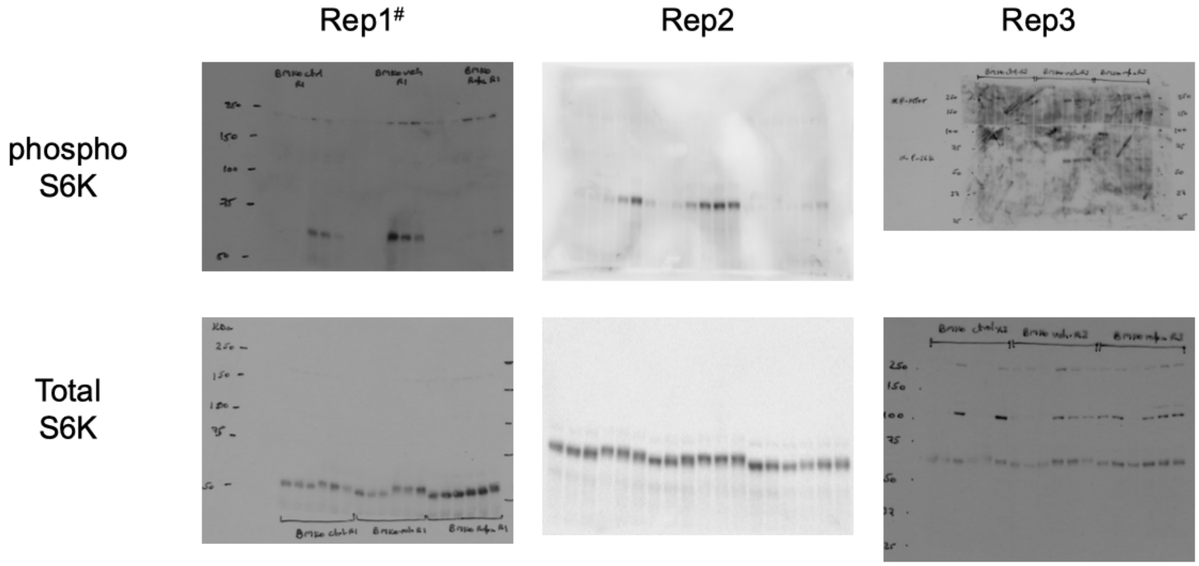

#Used in main figure

Figure S14

Western Blot from Fig. S2C

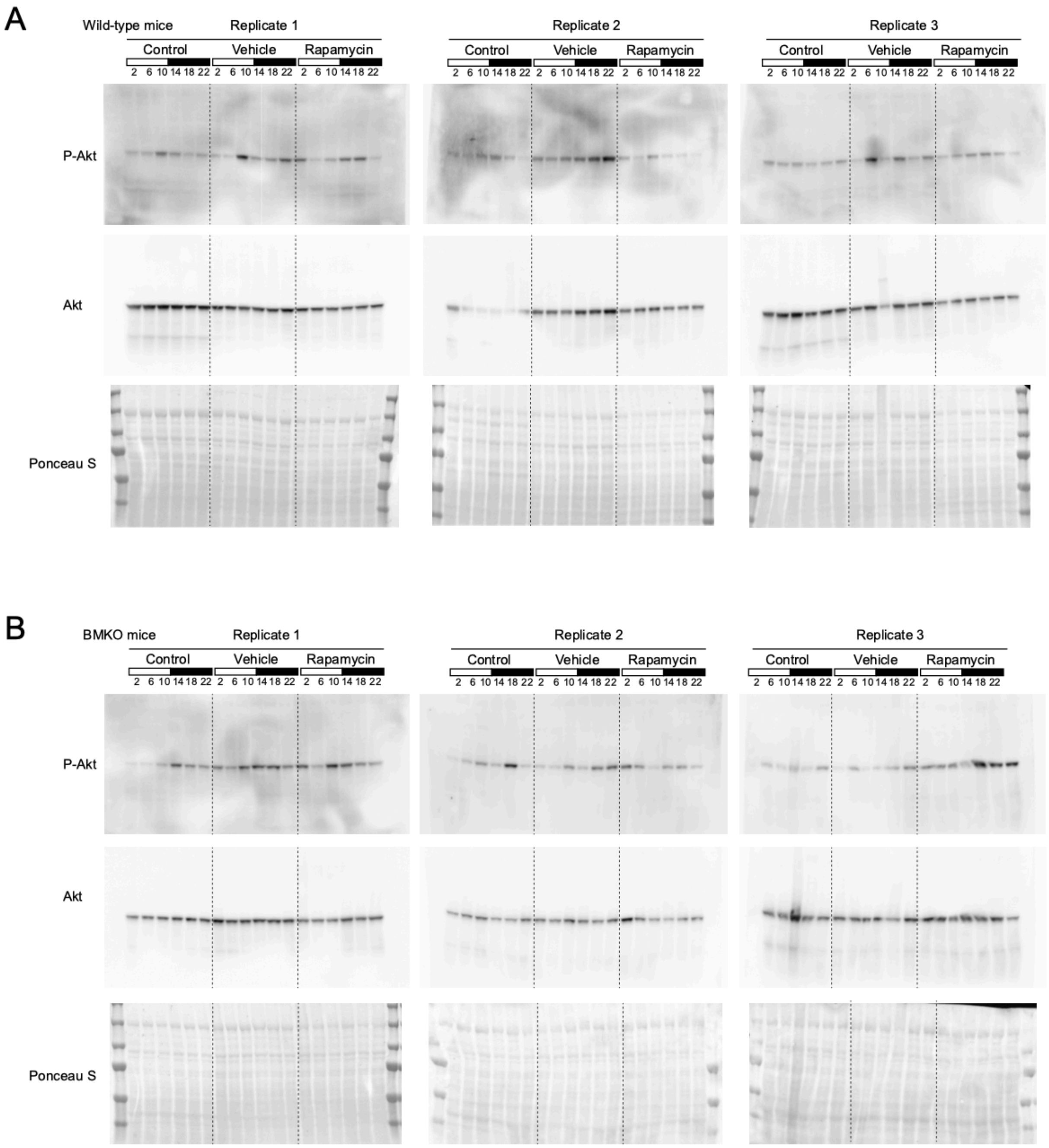

Figure S15

Western Blot from Fig. 2C and S5C

TSC1^{LKO}-NF

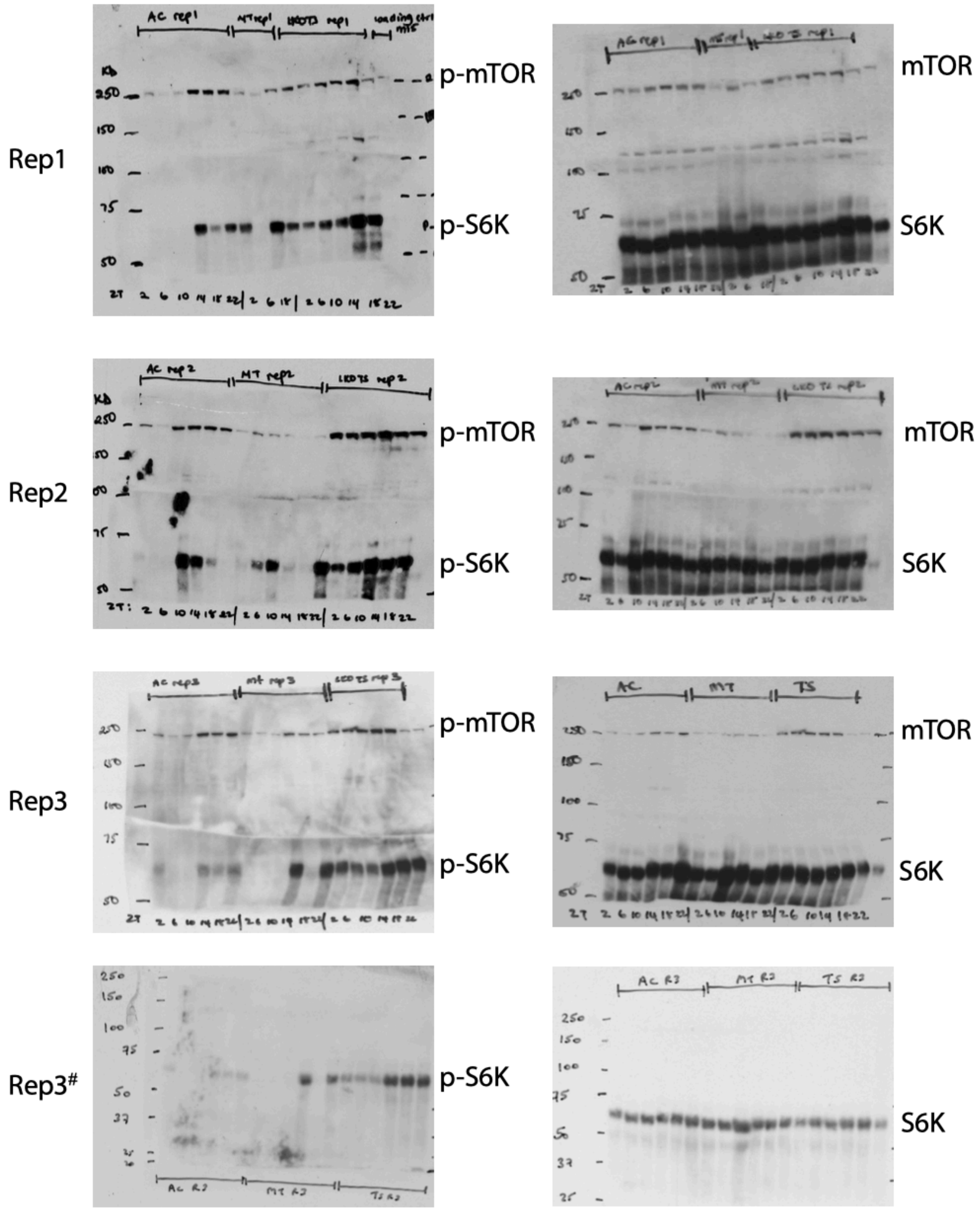

#Used in main figure

Figure S16

Western Blot from Fig. 2C, S5D, and S5F

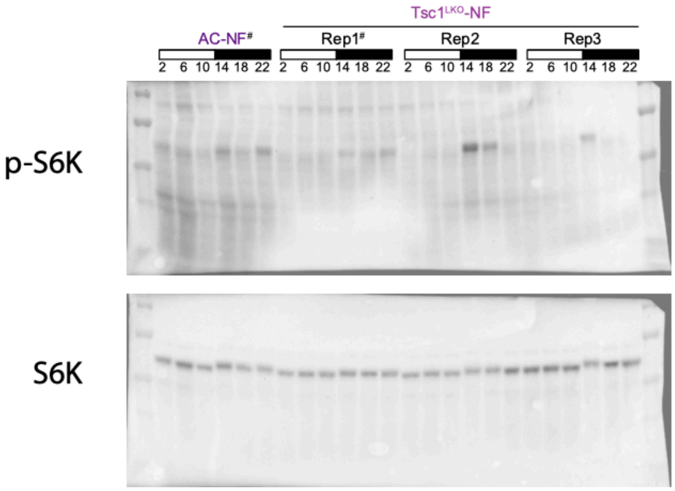

Western Blot from Fig. S2E

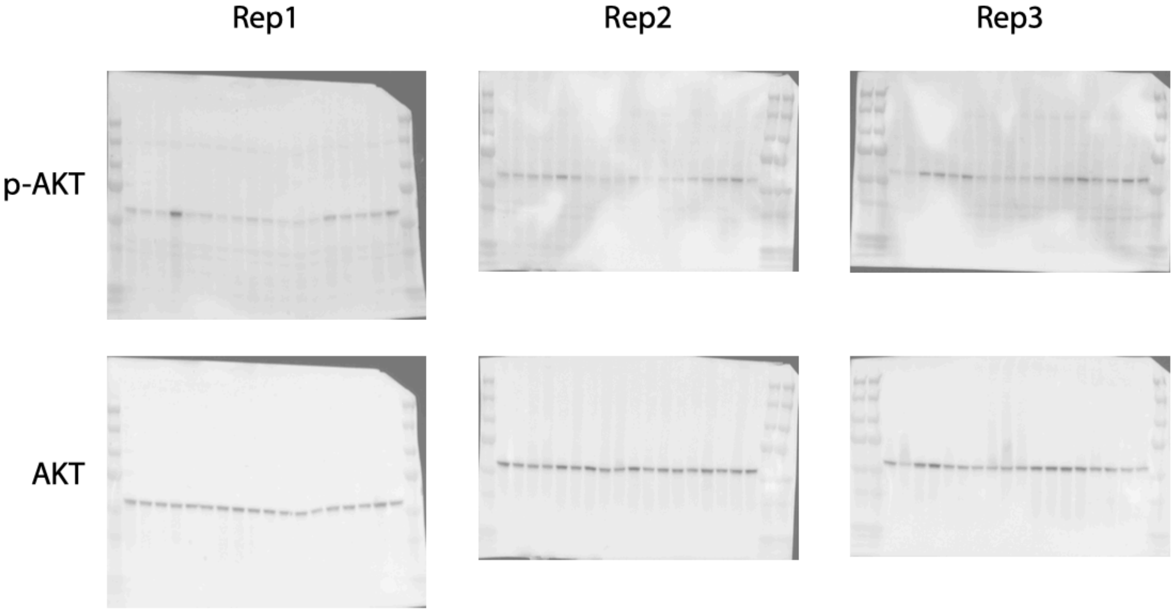

#Used in main figure

Figure S17

Western Blot from Fig. 3C and S6A

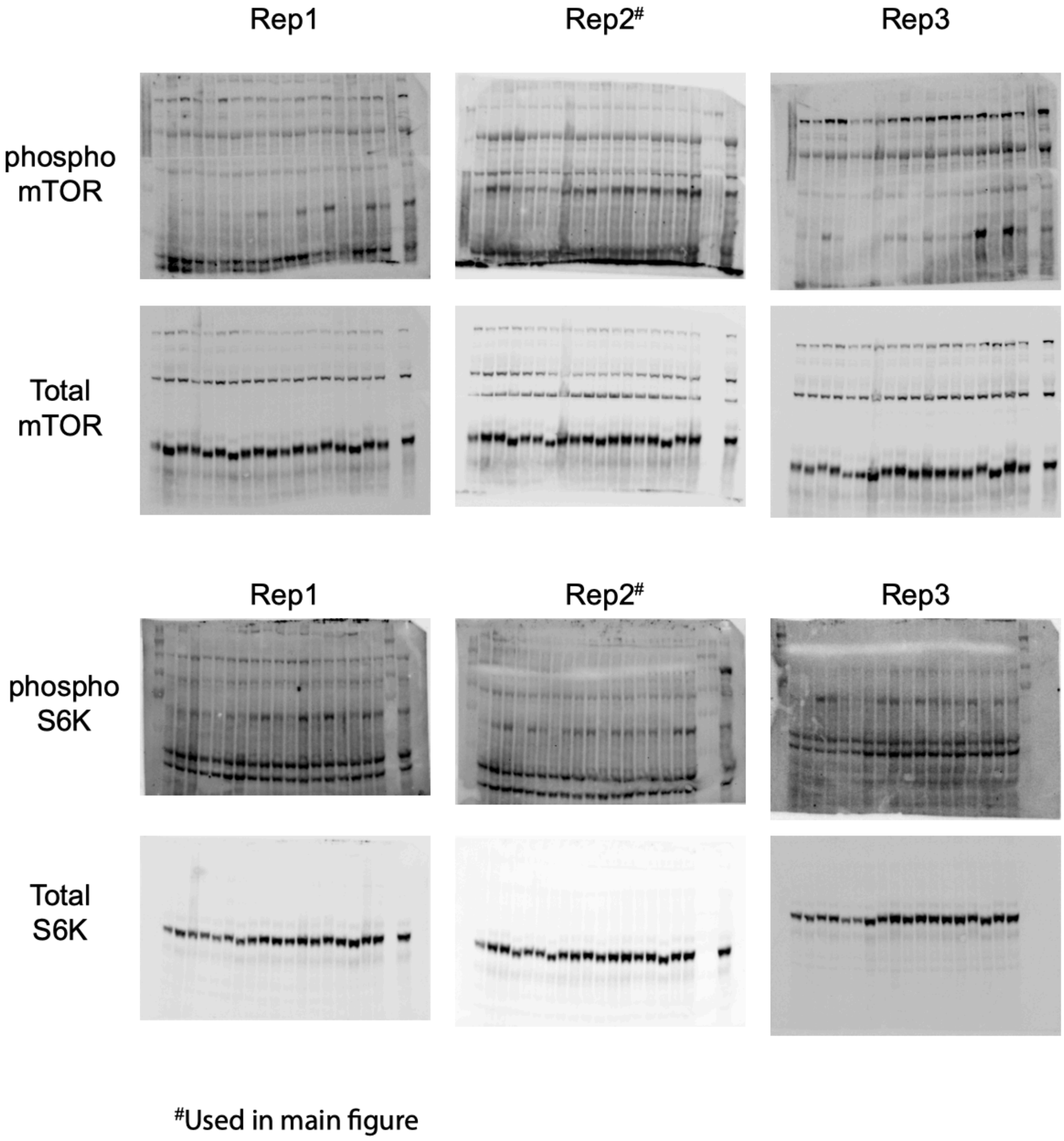

Figure S18

Western Blot from Fig. 6B and S9B

WT-NF-ctrl / WT-NF-veh / WT-NF-rapa

Rep1 Rep2 Rep3#

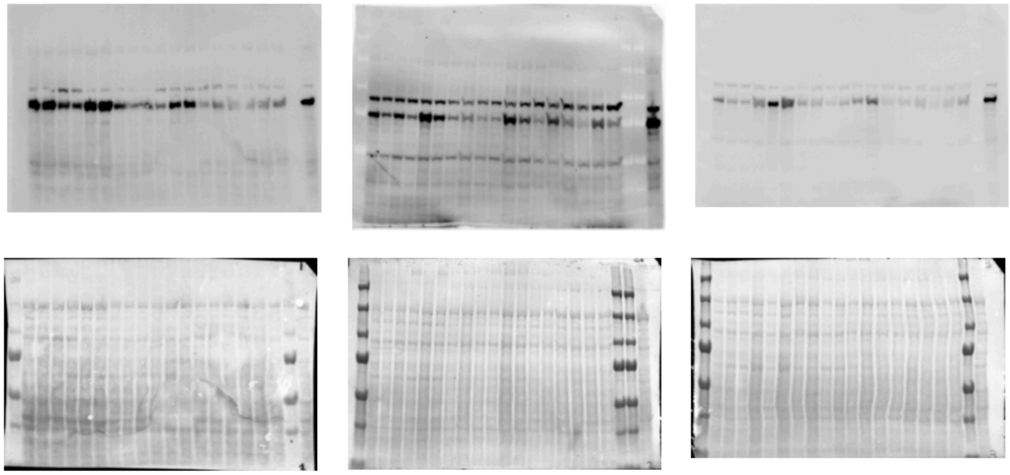

WT-AF / WT-AF_AZDday / WT-AF_AZDnight

Rep1# Rep2 Rep3

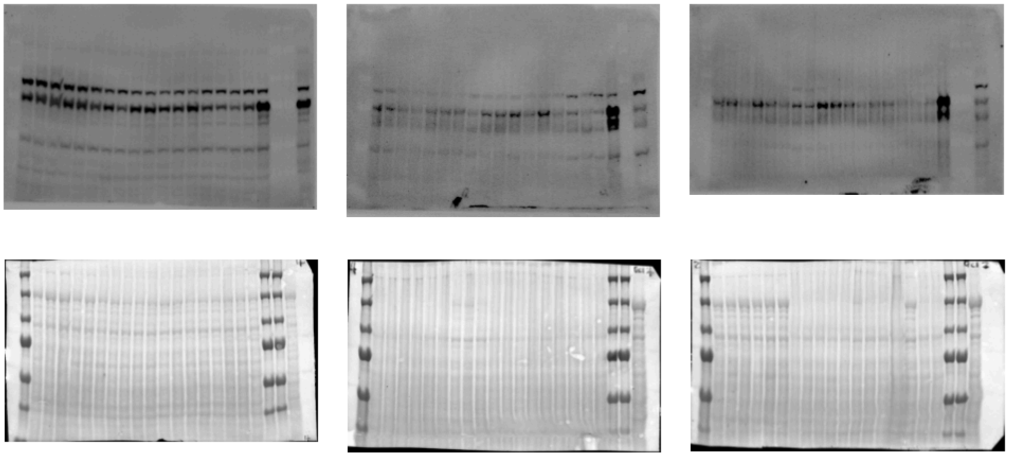

#Used in main figure

Figure S19

Western Blot from Fig. S10B

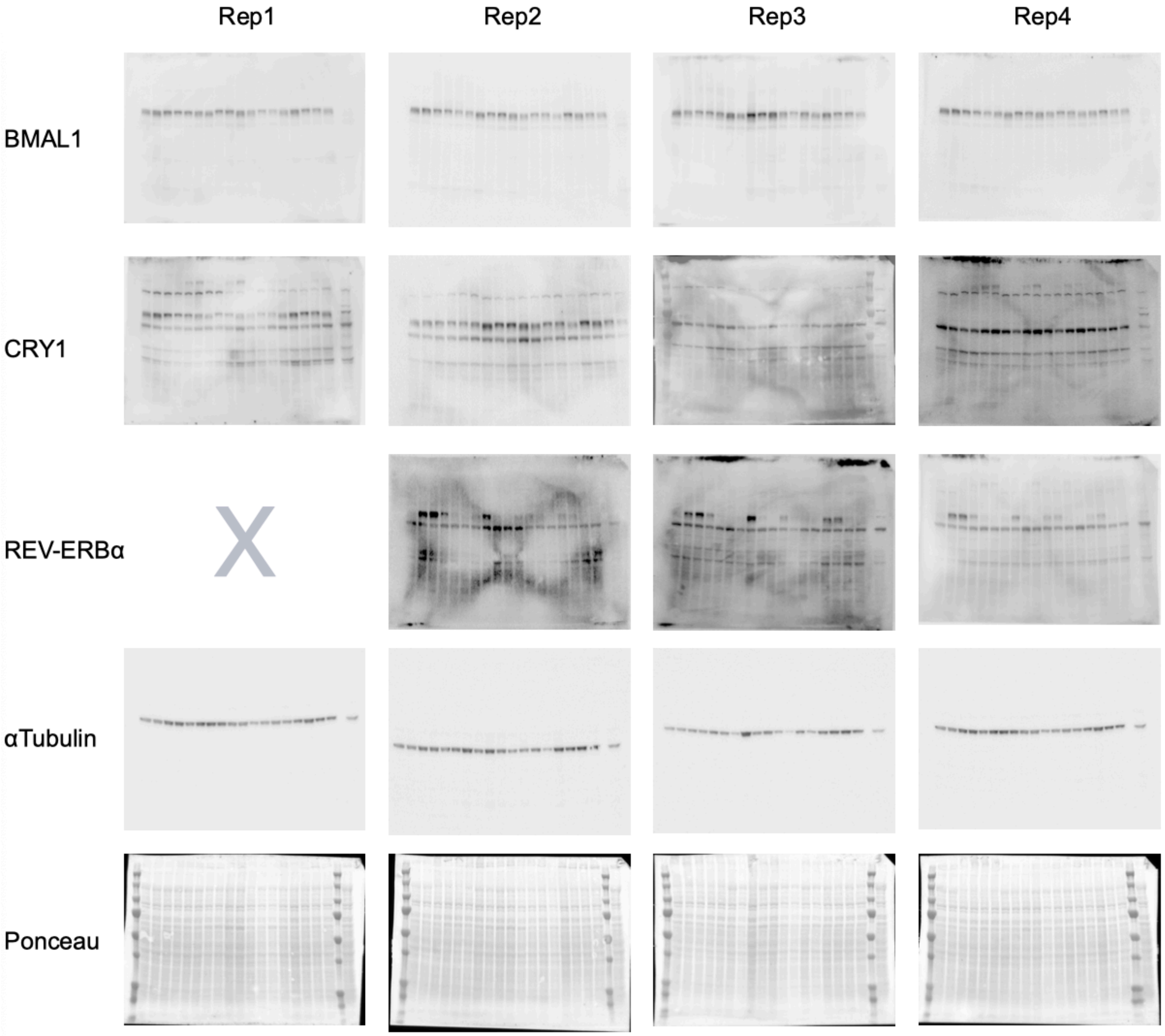

Figure S20

Supplementary Tables:

Table S1: Mouse liver RNA-Seq signal from all 13 rhythms.

Each rhythm consists of 6 time points, with n = 3 per time point. All names of mice formatted as $1_$2_$3_$4_$5, where:

$1 = Genotype (e.g., wild-type or KO mice)

$2 = Feeding parameter (NF=nighttime feeding; LB=ad libitum; AF=arrhythmic feeding)

$3 = Condition (Control, Rapa=rapamycin, Veh=vehicle, AZDday or AZDnight, TM=tunicamycin, or type of KO)

$4 = time point (ZT02, ZT06, ZT10, ZT14, ZT18, or ZT22; for 2, 6, 10, 14, 18, or 22 hours after light on)

$5 = replicate number (rep1, rep2, or rep3).

Table S2: Rhythmicity p-values for all 13 rhythms.

This table contains the p-values from JTK-cycle, F24, RAIN, and Harmonic regression algorithms, along with the harmonic average of the 4 p-values (hmp) and the Benjamini-Hochberg corrected hmp for each group and gene.

Table S3: Rhythmicity parameters for all 13 rhythms.

This table contains the relative amplitude (meta2d_rAMP; calculated with Metacycle algorithm), the F24 score (calculated with the F24 algorithm), the phase of expression (calculated with Harmonic Regression algorithm), the amplitude (calculated as Max - Min), the mean expression, the median expression, the relative amplitude (rAMP, calculated as amplitude / mean expression), and fold-change of expression (calculated as Max / Min) for each group and gene.

Table S4: KEGG pathway analysis of rhythmic genes for all 13 rhythms.

Results of the KEGG pathway enrichment analysis performed with the Limma package. EnrichGenes: number of genes from a pathway found rhythmic for this group; pval: p-value of enrichment.

Table S5: List of clock-driven and mTOR-driven rhythmic genes.

G1: clock-driven genes (C); G2: mTOR-driven rhythmic genes (M); G3: clock- or mTOR-driven rhythmic genes (CorM); G4: clock- and mTOR-driven rhythmic genes (CandM). s: stringent list; all: complete list.

Table S6: Motif analysis for the 4 groups of rhythmic genes.

Results for the motif analysis performed with the Homer package. G1: clock-driven rhythmic genes; G2: mTOR-driven rhythmic genes; G3: clock- or mTOR-driven rhythmic genes; G4: clock- and mTOR-

driven rhythmic genes. A: all genes; B: strong genes; C: remaining genes. The number of CRE analyzed for each group is: 602 for G1A; 414 for G1B; 188 for G1C; 751 for G2A; 369 for G2B; 382 for G2C; 1524 for G3A; 1063 for G3B; 461 for G3C; 766 for G4A; 219 for G4B; 547 for G4C; and 20000 for all CREs (background/control).

Table S7: List of mouse liver TF ChIP-Seq datasets reanalyzed in this study.

Table S8: Mouse RNA-Seq signal after tunicamycin injection

Each rhythm consists of 4 time points, with n = 4 per time point. All names of mice formatted as $1_$2_$3_$4_$5, where:

$1 = Genotype (e.g., WT=wild-type)
$2 = Feeding parameter (NF=nighttime feeding; AF=arrhythmic feeding)
$3 = Condition (TM=tunicamycin)
$4 = time point (ZT04, ZT10, ZT16, or ZT22; for 4, 10, 16, or 22 hours after light on)
$5 = replicate number (rep1, rep2, rep3, or rep4).

Table S9: Rhythmicity analysis of the tunicamycin RNA-Seq datasets

This table contains the p-values from JTK-cycle, F24, RAIN, and Harmonic regression algorithms, along with the harmonic average of the 4 p-values (hmp) and the Benjamini-Hochberg corrected hmp for each group and gene. It also contains the relative amplitude (meta2d_rAMP; calculated with Metacycle algorithm), the F24 score (calculated with the F24 algorithm), the phase of expression (calculated with Harmonic Regression algorithm), and the amplitude (calculated by Harmonic regression) for each group and gene.

Table S10: Raw values for metabolomics analysis in mouse liver

Tables containing original output of the mouse liver metabolomics study.

Table S11: Analysis of the mouse liver metabolome and rhythmicity analysis output

Table containing the 823 metabolites analyzed in this study and their levels across the 24-hour day in WT-NF-ctrl, WT-AF, and WT-AF-AZDnight mice. Each rhythm consists of 6 time points, with n = 3 biological replicate per time point. This table contains the rhythmicity p-values from ARSER, JTK-cycle, LS, F24, RAIN, and Harmonic regression algorithms, along with the harmonic average of the p-values (hmp) and the Benjamini-Hochberg corrected hmp for each group and gene. It also contains the relative amplitude (meta2d_rAMP; calculated with Metacycle algorithm), the F24 score (calculated with the F24

algorithm), the phase of expression (calculated with Harmonic Regression algorithm), and the amplitude (calculated by Harmonic regression) for each group and gene.
